# Protecting the Achilles heel: three FolE_I-type GTP-cyclohydrolases needed for full growth of metal-resistant *Cupriavidus metallidurans* under a variety of conditions

**DOI:** 10.1128/jb.00395-23

**Published:** 2024-01-16

**Authors:** Vladislava Schulz, Diana Galea, Martin Herzberg, Dietrich H. Nies

**Affiliations:** 1 Molecular Microbiology, Martin-Luther-University Halle-Wittenberg, Halle/Saale, Germany; 2 Department of Analytical Chemistry, Helmholtz Centre for Environmental Research – UFZ, Leipzig, Germany; University of Florida Department of Microbiology and Cell Science, Gainesville, Florida, USA

**Keywords:** folate biosynthesis, zinc, ZMP

## Abstract

In *Cupriavidus metallidurans* and other bacteria, biosynthesis of the essential biochemical cofactor tetrahydrofolate (THF) initiates from guanosine triphosphate (GTP). This step is catalyzed by FolE_I-type GTP cyclohydrolases, which are either zinc-dependent FolE_IA-type or metal-promiscuous FolE_IB-type enzymes. As THF is also essential for GTP biosynthesis, GTP and THF synthesis form a cooperative cycle, which may be influenced by the cellular homeostasis of zinc and other metal cations. Metal-resistant *C. metallidurans* harbors one FolE_IA-type and two FolE_IB-type enzymes. All three proteins were produced in *Escherichia coli*. FolE_IA was indeed zinc dependent and the two FolE_IB enzymes metal-promiscuous GTP cyclohydrolases *in vitro*, the latter, for example, functioning with iron, manganese, or cobalt. Single and double mutants of *C. metallidurans* with deletions in the *folE_I* genes were constructed to analyze the contribution of the individual FolE_I-type enzymes under various conditions. FolE_IA was required in the presence of cadmium, hydrogen peroxide, metal chelators, and under general metal starvation conditions. FolE_IB1 was important when zinc uptake was impaired in cells without the zinc importer ZupT (ZIP family) and in the presence of trimethoprim, an inhibitor of THF biosynthesis. FolE_IB2 was needed under conditions of low zinc and cobalt but high magnesium availability. Together, these data demonstrate that *C. metallidurans* requires all three enzymes to allow efficient growth under a variety of conditions.

**IMPORTANCE:**

Tetrahydrofolate (THF) is an important cofactor in microbial biochemistry. This “Achilles heel” of metabolism has been exploited by anti-metabolites and antibiotics such as sulfonamide and trimethoprim. Since THF is essential for the synthesis of guanosine triphosphate (GTP) and THF biosynthesis starts from GTP, synthesis of both compounds forms a cooperative cycle. The first step of THF synthesis by GTP cyclohydrolases (FolEs) is metal dependent and catalyzed by zinc- or metal-promiscuous enzymes, so that the cooperative THF and GTP synthesis cycle may be influenced by the homeostasis of several metal cations, especially that of zinc. The metal-resistant bacterium *C. metallidurans* needs three FolEs to grow in environments with both high and low zinc and cadmium content. Consequently, bacterial metal homeostasis is required to guarantee THF biosynthesis.

## INTRODUCTION

The metal-resistant beta-proteobacterium *Cupriavidus metallidurans* possesses a remarkable ability to cope with zinc concentrations from the nanomolar to the lower millimolar range (1, 2). This enables it to survive high concentrations of zinc and other transition metal cations (2) but surprisingly also to manage zinc starvation conditions (3–5). *C. metallidurans* inhabits environments such as zinc deserts and auriferous soils (6–9).

The genes for zinc transport and handling are located on the bacterial chromosome, a chromid, and two large plasmids (pMOL28 and pMOL30) in *C. metallidurans* strain CH34 wild type (2, 10). Zinc ions are imported into the cytoplasm of *C. metallidurans* by at least nine import systems (11–14), but only ZupT of the ZIP protein family [TC#2.A.5 (15, 16)] is controlled by intracellular zinc availability (11–14). The Zur zinc uptake regulator of the Fur protein family represses *zupT* expression in the presence of Zn(II) (5, 17–22). Additional members of the Zur regulon in *C. metallidurans* are three genes (*cobW1, 2, 3*) for GTPases of the COG05203 protein family (5, 17, 23, 24). A member of this family in *Bacillus subtilis* has recently been re-named “ZagA” for “ZTP-activated GTPase A” (25).

The gene for CobW1 is part of the *cobW1*-operon Op0317f_1 (Fig. S1), which is needed for full resistance to metal starvation conditions (4, 26). While expression of the genes for most members of the Zur regulon in *C. metallidurans* possesses only one Zur-binding box in the promoter region as regulatory element, with the consequence that their products are also present in cells cultivated in the presence of 200 nM Zn(II) in the growth medium (27), two Zur boxes are present in upstream of Op0317f_1. These genes are only expressed under zinc starvation conditions, at about 50 nM Zn(II) or below (5, 17, 27), or in the absence of the Zur repressor (5, 17, 27).

The operon Op0317f_1 (Fig. S1) contains in addition to *cobW1* genes for the GTP cyclohydrolase FolE_IB2 (Rmet_1099), the Cys-tRNA-synthetase CysS2, QueD involved in queuosine biosynthesis, which is also initiated by the FolE-dependent GTP cyclohydrolyzation, the allantoinase AllB, and a carbonic anhydrase (4). While all these enzymes are probably zinc dependent, *folE_IB2* encodes a GTP cyclohydrolase that may not be strictly zinc dependent but metal promiscuous instead (28). *C. metallidurans* possesses two additional FolE-type enzymes. The predicted zinc-dependent FolE_IA (Rmet_3990) is encoded on the bacterial chromid and a second possible metal promiscuous FolE_IB1 (Rmet_2614) on the bacterial chromosome (Fig. S1). The metal cofactor acts as Lewis acid, binding a hydroxyl ion, which attacks the imidazole ring of the substrate GTP (29).

**Fig 1:**
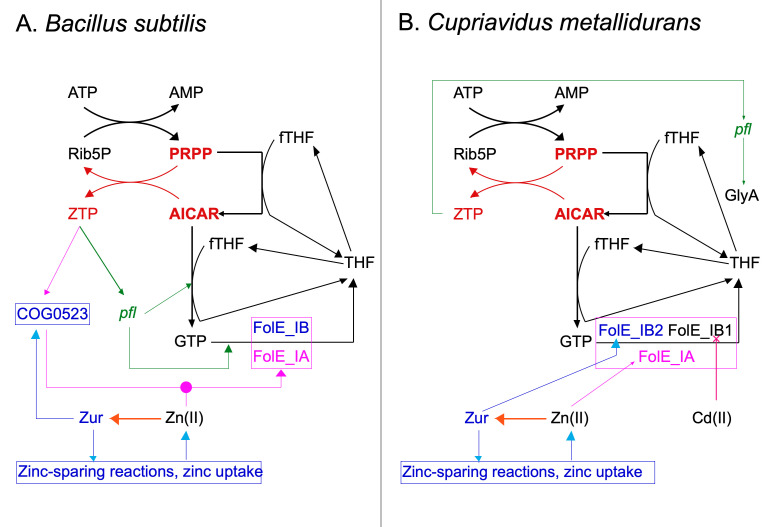
ZTP regulatory circuits are shown in for *Bacillus subtilis* (panel A) and for *C. metallidurans* (panel B). Black: GTP is a product of the bacterial purine biosynthesis pathway, which starts with an ATP-dependent pyrophosphorylation of ribose-5-phosphate (Rib5P) to 5-phosphoribosyl-1-pyrophosphate (PRPP) and continues via inosine monophosphate (not shown) and guanosine monophosphate to GTP. Two of the biochemical reactions on this pathway depend on N^5^,N^10^-methenyl- or N^10^-formyl-tetrahydrofolate. The latter transforms 5′-phosphoribosyl-4-carboxyamide-5-aminoimidazole (AICAR or ZMP) into IMP and is catalyzed by the bi-functional enzyme PurH. The THF biosynthesis pathway starts with GTP-cyclohydrolases, which could belong to the zinc-dependent FolE_IA-type or the metal-cambialistic FolE_IB-type enzymes. Tetrahydrofolate is loaded with C1 groups, for instance, by the glycine hydroxymethylase, which splits serine into glycine and fTHF (30). Red: During THF starvation, transformation of AICAR to IMP by the strictly fTHF-dependent PurH enzyme is reduced, AICAR accumulates and is pyrophosphorylated by PRPP to ZTP. Green: Using the pfl riboswitch, ZTP activates operons involved in purine, THF biosynthesis, and/or interconversion of THF derivatives, such as fTHF, to compensate for fTHF starvation; different genes are used in different bacteria. An increased transformation of AICAR to IMP, GTP, and THF may cause as a back-reaction ZTP-dependent pyrophosphorylation of Rib5P to AICAR, consuming ZTP and switching-off the response. In *C. metallidurans, pfl* is upstream of *glyA*. Blue: Under conditions of cellular zinc sufficiency, the Zur regulator is activated by Zn(II) and prevents expression of genes involved in zinc uptake and consumption. These Zur-controlled operons are de-repressed when zinc ions are lacking in the cytoplasm (orange arrow). Some of these genes encode proteins of the COG0523 protein group such as the CobW proteins in *C. metallidurans*, which are zinc chaperones, some FolE_IB-type GTP-cylohydrolases that do not strictly depend on zinc for activity. Purple: This sophisticated ZTP-dependent regulation of purine and THF biosynthesis is also exploited in *B. subtilis* to influence zinc homeostasis by recruiting the THF-influenced AICAR pool as sensor for zinc availability. If not compensated by the FolE_IB, decreased activity of the zinc-dependent FolE_IA enzyme due to zinc under-metallation leads to a diminished THF concentration, reduced activity of the fTHF-dependent PurA enzyme, and accumulation of AICAR and ZTP. A publication by the Helmann group (25) demonstrates that the COG0523 protein YciC from *B. subtilis* is activated by ZTP, which may result in an enhanced delivery of the zinc to FolEIA. Consequently, YciC was renamed “ZTP-activated GTPase A,” ZagA. This connection has not been demonstrated in *C. metallidurans*. This publication investigates the influence of the three FolE_I-type enzymes in *C. metallidurans* and the influence of cadmium. Figure taken from reference (32) with permission.

When tetrahydrofolate (THF) biosynthesis is disturbed due to a lack of FolE_I-type enzyme activity, production of methionine, formyl-methionine-tRNA for translation initiation, and dTTP for DNA replication may be diminished (Fig. 1). Moreover, THF biosynthesis starts with cyclohydrolyzation of GTP, but GTP biosynthesis depends on the PurH-catalyzed N^10^-formyl-THF (fTHF)-dependent conversion of AICAR (5′-phosphoribosyl-4-carboxyamide-6-aminoimidazole, Z-nucleotide monophosphate, ZMP) to inosine monophosphate (IMP), which is transformed in two steps mediated by GuaB and GuaA to GMP. While synthesis of AICAR is possible without THF as cofactor due to the PurT-mediated reaction and, additionally, as by-product of the *de novo* histidine biosynthesis, further conversion of AICAR to IMP, GMP, and finally to GTP strictly requires THF (30–32). This leads to a negative cooperative cycle; no THF without GTP and no GTP without THF. It is in this way that THF- and zinc-starvation responses are interconnected in the firmicute *B. subtilis* via a zinc-dependent FolE_I enzyme (25).

Here, we studied the three FolE_I-type enzymes and their physiological function as possible “Achilles heels” of a disturbed metal homeostasis in *C. metallidurans* to understand why a zinc-resistant bacterium needs two extra FolE_I-type enzymes in addition to the putative zinc-dependent FolE_IA. We found that FolE_IA is zinc dependent but malfunctions under zinc starvation conditions, while the two FolE_IB enzymes are metal promiscuous but sensitive to cadmium and hydrogen peroxide stress. So, under cadmium stress, the FolE_IBs are inhibited, but sufficient zinc is still available for FolE_IA. Under metal starvation, FolE_IA is not fully functional, but the FolE_IBs can take over THF biosynthesis. In this way, sufficient THF is always guaranteed to be available in metal-resistant *C. metallidurans*.

## RESULTS

### Enzymatic activities

The three FolE-type enzymes FolE_IA (locus tag Rmet_3990), FolEI_IB1 (Rmet_2614), and FolE_IB2 (Rmet_1099) from *C. metallidurans* were produced in *Escherichia coli* as N-terminal (FolE_IA) or C-terminal (FolE_IB’s) Strep-tag fusion proteins and purified by affinity chromatography (Fig. S2 through S4). As determined by inductively coupled plasma mass spectrometry (ICP-MS), FolE_IA contained 0.6 Zn per mol protomer (Table 1). The specific activity of FolE_IA as GTP cyclohydrolase was determined in a photometric assay at 330 nm, which measured product formation to be 41 U/g protein (Table 1). The activity of FolE_IA could not be inhibited by 5 mM EDTA, treatment with 80 µM TPEN (20-fold excess), 100 µM Co(II), or 1 mM Mn(II) but could be inhibited to 40% by 2 mM Mn(II) (Table 2). After incubation of non-catalyzing FolE_1A with 20 µM Cd(II) followed by buffer exchange, FolE_IA was >99% inhibited, but its activity could be increased fivefold again by the addition of 50 µM Zn(II) (Table 2). FolE_IA was a zinc-containing enzyme, its activity could not be stimulated by the addition of Zn(II), Co(II), Mn(II), or Cd(II), and the zinc ion did not exchange freely with zinc-complexing compounds, indicating firm binding of the cofactor to the enzyme. Cadmium inhibited non-catalyzing FolE_IA, but addition of zinc restored the activity to some part again.

**TABLE 1:** Specific activity of FolE-type enzymes from *C. metallidurans^a^*

	FolE_IA	FolE_IB1	FolE_IB2
Molecular mass, kDa	31	32	38
Position Strep-tag	N-terminal	C-terminal	C-terminal
Specific activity, U/g	40.8 ± 2.1	0	0
Metal content (ICP-MS), mol/mol, holo-form	0.60 ± 0.03 Zn Fe b.d.l.^*b*^	0.10 ± 0.01 Zn 0.36 ± 0.14 Fe	0.06 ± 0.01 Zn 0.82 ± 0.06 Fe
Metal content (ICP-MS), mol/mol, apo-form	n.d.^*d*^	0.04 ± 0.04 Zn 0.04 ± 0.05 Fe	0.08 ± 0.01 Zn 0.27 ± 0.12 Fe
Apo-form + 1 mM Fe(II)^*c*^ (ICP-MS), mol/mol	n.d.	18.4 ± 0.2.2 Fe	63.2 ± 0.5 Fe

^*a*^ The enzymes were produced in *Escherichia coli* and purified by affinity chromatography. The specific activity and metal content as determined by ICP-MS measurements are provided for the resulting holo-enzyme. For the FolE_IBs, an apo-form could be obtained by treatment with EDTA.

^*b*^ Fe b.d.l, below detection limit.

^*c*^ Anaerobically reconstituted with Fe(II).

^*d*^ n.d., not determined.

**TABLE 2:** Activity of FolE_IA in the presence of metal ions or chelators^*a*^

Addition	FolE_IA^*b*^	c_FolE_IA_ assay (µM)
Directly added to the assay		
None	100% ± 5%	2
5 mM EDTA	103% ± 0%	2
50 µM Zn(II)	79% ± 2%	2
50 µM Zn(II) after pre-incubation with 20 µM Cd(II)	3.3% ± 0.4%	2
100 µM Co(II)	92% ± 7%	2
1 mM Mn(II)	92% ± 9%	2
2 mM Mn(II)	58% ± 7%	2
Pre-incubated^*b*^		
80 µM TPEN	85% ± 10%	4
20 µM Cd(II)	0.68% ± 0.02%	2

^*a*^ The specific activity was determined in the GTP-cyclohydrolase assay using 2 or 4 µM FolE_IA (holo-form) and divided by the specific activity of the control without addition. Specific activity of FolE_IA without addition of 40.8 ± 2 U/g, *n* ≥3, deviations given.

^*b*^ And 4 µM of FolE_IA (holo-form) was mixed with TPEN or Cd(II) and incubated on ice for 2 h. Afterward the buffer was exchanged by desalting column, and the enzyme was applied as described for the assay after the concentration had been determined.

In contrast to FolE_IA, neither FolE_IB1 nor FolE_IB2 displayed any enzymatic activity when isolated from *E. coli* cells (Table 1). Both proteins contained iron: FolE_IB1 0.36 mol/mol and FolE_IB2 0.82 mol/mol (Table 1). Treatment of these holo-forms of the two FolE_IB-type proteins with EDTA generated apo-forms, which no longer contained significant amounts of iron in the case of FolE_IB1 and one-third of the iron content of the holo-form in the case of FolE_IB2 (Table 1). In the presence of manganese, all four proteins acquired cyclohydrolase activity: 4.6 U/g for holo-FolE_IB1 and 6.2 U/g for its apo-form; 1.9 U/g for holo-FolE_IB2 and 2.2 U/g for its apo-form (Table 3). Holo-FolE_IB1 could even be activated by Mg(II) and Ni(II), but its apo-form could not be activated. Neither form of FolE_IB2 could be activated by Mg(II) and the holo-form of FolE_IB1 not by Zn(II) (Table 3).

**TABLE 3:** Activation of FolE_IB1 and FolE_IB2^*a*^

Enzyme	Holo-FolE_IB1		Apo-FolE_IB1		Holo-FolE_IB2		Apo-FolE_IB2	
Metal	(mM)	U/g pr.	(mM)	U/g pr.	(mM)	U/g pr.	(mM)	U/g pr.
Mn(II)	4.0	4.6 ± 0.4	4.0	6.2 ± 0.6	2.0	1.9 ± 0.4	4.0	2.2 ± 0.8
Co(II)	0.25	1.5 ± 0.1	0.05	5.4 ± 0.6	0.05	0.3 ± 0.1	0.05	1.2 ± 1.0
Ni(II)	0.5	0.3 ± 0.0	0.05	0.1 ± 0.0	n.d.^*c*^		n.d.	
Mg(II)	4.0	1.0 ± 0.2	1.0	0.1 ± 0.1				
Zn(II)	0.1	0.2 ± 0.2	n.d.					
Fe(II)^*b*^	0.5	7.2 ± 0.8	0.5	23.8 ± 3.2	0.5	6.1 ± 2.4	0.5	22.2 ± 2.2

^*a*^ The GTP-cyclohydrolase assay was performed with 4 µM purified enzyme (holo- and apo-forms of FolE_B1 and FolE_IB2) in the presence of increasing Mn(II), Mg(II), Fe(II) or Co(II), Ni(II), and Zn(II) concentrations. The specific activity in U/g protein was determined. The highest activity obtained with a specific metal ion and concentration was listed. Apo-FolE_IB2 could not be activated with Mg(II).

^*b*^ Incubation with Fe(II) was performed under anaerobic conditions. The enzymes were inhibited at higher metal concentrations, precipitated at 1 mM Fe(II), and started to precipitate at 0.5 mM Fe(II) in case of the two apo-forms. All reconstitutions under oxic conditions *n* >3, anaerobic reconstitution of FolE_IB1 *n* = 2, of FolE_IB2 *n* = 3, deviations shown.

^*c*^ n.d., not determined.

Co(II) also activated these enzymes (Table 3) with the apo-forms being activated to a much higher specific activity compared to the respective holo-forms: 5.4 U/g at 50 µM Co(II) for apo-FolEI_B1 versus 1.5 U/g for the holo-form and 1.2 U/g for apo-FolE_IB2 at 50 µM Co(II) versus 0.3 U/g for holo-FolE_IB2. Removal of the residual Fe from the FolE_IBs resulted in a better activation by Co(II) (Table 3).

Under anoxic conditions, the holo- and apo-forms of both FolE_IBs could also be activated by Fe(II), reaching 7.2 U/g and 6.1 U/g for holo-FolE_IB1 and holo_IB2, respectively, and even 24 U/g and 22 U/g for the two apo-forms (Tables 1 and 3), which was half of the specific activity of FolE_IA. This indicated that the two FolE_IBs were iron-dependent enzymes that were also able to function with manganese or cobalt ions. FolE_IB1 reached a similar specific activity with Mn or Co than FolE_IB2 (Table 3).

The activities of zinc-dependent FolE_IA and holo-FolE_IB1 plus 2 mM Mn(II) were studied in the presence of Cd(II) and H_2_O_2_ (Table 4). Activity of both enzymes decreased with increasing inhibitor concentrations. The enzyme activity of FolE_IA was more stable than FolE_IB1 in the presence of both substances. Cadmium inhibited FolE_IA at 200 µM to 81%, but even in the presence of this cadmium concentration, the activity of FolE_IA was still 7.4 U/g. FolE_IA was more stable in the presence of cadmium when it was enzymatically active for 2 h at 30°C compared to a situation when the non-catalyzing protein was challenged with cadmium on ice for the same length of time. Zinc could partially restore the enzymatic activity of the cadmium-damaged FolE_IA (Table 2). Under conditions resembling those *in vivo*, FolE_IA displayed considerable cadmium resistance.

**TABLE 4:** Comparison of the activity of FolE_IA and FolE_1B1 in the presence of cadmium and hydrogen peroxide^*a*^

Addition	FolE_IA		FolE_IB1^*b*^	
	U/g	%	U/g	%
None^*b*^	38.5 ± 7.6	100% ± 20%	4.3 ± 0.9	100% ± 21%
10 µM Cd(II)	25.0 ± 1.3	65% ± 3%	3.3 ± 0.3	75% ± 7%
40 µM Cd(II)	18.1 ± 3.3	47% ± 9%	2.5 ± 0.4	58% ± 10%
0.1 mM Cd(II)	14.4 ± 2.6	37% ± 7%	0.8 ± 0.1	18% ± 2%
0.2 mM Cd(II)	7.4 ± 1.9	19% ± 5%	0.3 ± 0.8	8% ± 18%
1 mM H_2_O_2_	31.3 ± 3.6	81% ± 9%	2.7 ± 0.3	61% ± 6%
2 mM H_2_O_2_	24.9 ± 7.4	65% ± 19%	1.3 ± 0.5	30% ± 12%
10 mM H_2_O_2_	21.1 ± 4.6	55% ± 12%	0.2 ± 0.1	5% ± 2%
20 mM H_2_O_2_	13.3 ± 3.3	35% ± 9%	−0.1 ± 0.1	−1% ± 3%

^*a*^ The specific activity was determined in the GTP-cyclohydrolase assay using 4 µM enzyme and divided by the specific activity of the control without addition. These were the holo-forms of FolE_IA as isolated.

^*b*^ FolE_B1 in the presence of 2 mM Mn(II) to activate the enzyme, *n* ≥3, deviations given.

In contrast, inhibition of FolE_IB1 was nearly complete at 200 µM cadmium. Likewise, FolE_IA was inhibited by 65%, but FolE_IB1 was completely inhibited at 20 mM H_2_O_2_. FolE_IA hydrolyzed GTP even at high cadmium and H_2_O_2_ concentrations, while FolE_IB1 did not (Table 4). This indicated that FolE_IB1 was much more sensitive to cadmium and reactive oxygen species (ROS) than FolE_IA.

These biochemical experiments indicated that FolE_IA was a zinc-dependent enzyme. Its activity was more stable in the presence of cadmium or ROS than that of FolE_IB1. The specific activity of FolE_IA was two times higher than the highest activity measured for FolE_IB1, while those of the two FolE_IBs were similar. Both FolE_IBs together reached the same activity level as FolE_IA. On the other hand, FolE_IB1 and FolE_IB2 could be activated by iron, manganese, cobalt, and to a small extend by nickel and magnesium. Cations could be removed by EDTA treatment of the FolE_IBs, and the resulting apo-forms re-loaded with different metallic cofactors. Based on these findings, we initiated studies to identify the physiological function of these enzymes in *C. metallidurans*.

### Regulation of expression of the *glyA* gene

Conversion of AICAR or ZMP (Fig. 1) to GTP strictly depends on fTHF, such that ZMP accumulates under fTHF starvation and can thus be phosphorylated to ZTP (25). The *pfl* riboswitch is a ZMP/ZTP-dependent riboswitch that triggers changes in expression in the genes downstream of *pfl* (33, 34). BLAST searches (35) identified one *pfl* in the *C. metallidurans* genome with 100% sequence identity to the 103-bp *pfl* sequence of *B. subtilis* (Fig. S5); the second-best scoring sequence displayed sequence identity for only 18 of 19 base pairs. *C. metallidurans* contained only one *pfl*-like sequence. It was located upstream of the *glyA* gene, encoding serine hydroxymethyltransferase (Fig. S5), which forms glycine and 5,10 methylene-THF. Glycine can be further degraded to form a second C1-THF moiety. In *C. metallidurans*, ZMP or ZTP may control the pathway required for loading of THF with C1 compounds by degradation of serine and subsequently of the resulting glycine (Fig. 1).

A *glyA-lacZ* operon fusion was constructed and introduced into several mutant backgrounds. Expression of *glyA-lacZ* was studied in the presence of inhibitors of THF biosynthesis, the dihydrofolate reductase inhibitor trimethoprim (TMP), and the dihydropteroate synthase inhibitor sulfonamide (SUAM), which influence the last and penultimate step in THF biosynthesis, respectively (36, 37). Activity of the reporter was up-regulated in strain AE104 by TMP, while SUAM had no effect (Table S1). This indicated that TMP caused folate starvation in *C. metallidurans*, which leads to a lack of fTHF. Moreover, the ZMP/ZTP-dependent riboswitch *pfl* was functional in *C. metallidurans* and controlled expression of its downstream gene *glyA* (Fig. 1). Consequently, *pfl-glyA-lacZ* could be used as a reporter to measure fTHF availability and the subsequent ZMP accumulation in *C. metallidurans*.

While the ∆*zupT* deletion had no effect on *glyA-lacZ* expression, the reporter activity was down-regulated in the ∆*zur* strain without any metal addition (Table S1). The constitutive expression of the Zur regulon components in the *C. metallidurans* ∆*zur* mutant (5, 17) mediated accelerated conversion of ZMP to GTP, as measured by a decreased expression of *pfl-glyA-lacZ*, indicating increased availability of fTHF and maybe also folate. Addition of zinc to strain AE104 had no effect on *pfl-glyA-lacZ* expression, not even when the gene for the metal-promiscuous FolE_B1 was deleted, so that folate synthesis depended solely on zinc-containing FolE_IA (Table S1). Addition of yeast extract as external folate source also had no effect on the growth of strain AE104, so that external folate did not seem to influence fTHF availability in the cells.

Deletion of *folE_IB1* increased *pfl-glyA-lacZ* expression; however, a *folE_IA* deletion did not. Additional deletion of *zupT*, encoding the zinc importer, caused a similar phenotype (Table S1). The cysteine content of yeast extract causes sequestration of all transition metal cations (27), decreasing their availability. The exception is Fe(III), which could by accumulated by the siderophore staphyloferrin B of *C. metallidurans* (38–40). Consequently, decreased availability of zinc for FolE_IA resulted in an increased importance of metal-promiscuous FolE_IB1 to produce sufficient folate and fTHF for efficient conversion of ZMP to GTP (Fig. 1).

### Regulation of expression of the *folE* genes

The gene *folE_IB2* was expressed as part of the operon Op0371f_1, which is under Zur control but only under zinc starvation conditions (Fig. S1C). The other two *folE* genes were expressed in cells cultivated under non-challenging conditions with NPKM values of 61 (*folE_IA*) and 331 (*folE_IB1*) from promoters that were not dependent on the house-keeping sigma factor RpoD, leading to a protein copy number of about 181 (69–293) FolE_IA and 512 (400–624) FolE_IB1 per cell (27), while no FolE_IB2 could be found under these conditions. In contrast to *folE_IB2,* the other two genes are not responsive to zinc starvation, or to surplus zinc or cadmium (Fig. S1).

Fusions with a promoter-less *lacZ* -gene were constructed for *folE_IA* and *folE_IB1*, leaving the respective native gene intact, and coupled expression of both genes was studied using this reporter system. There was no change in expression of *folE_IA-lacZ* or of *folE_IB1-lacZ* in the presence of SUAM or of TMP (Table S2), which caused folate starvation as indicated by the *plf-glyA-lacZ* fusion (Table S1). Additional deletion of *zupT* or *zur* had no effect on expression levels (Table S2). Both genes were not controlled by accumulation of ZMP caused by fTHF starvation.

There was no change in the expression of either gene when the metal chelators EDTA, DIP (dipyridyl or bipyridyl), or 1 µM *N*,*N*,*N′*,*N′*-tetrakis(2-pyridinylmethyl)-1,2-ethanediamine (TPEN) were added to the growth medium (Table S3). The exception was a barely significant decrease of reporter activity of the *folE_IB1-lacZ* fusion in the presence of dipyridyl (31% decrease, *D* = 1.12). Again, deletion of *zupT* or *zur* did not change the influence of the metal chelators on *folE_IA* or *folE_IB* expression.

Deletion of the respective other gene led to a nearly twofold up-regulation of *folE_IB1-lacZ* expression in the ∆*folE_IA* mutant and to an up-regulation of about 25% of the ∆*folE_IA-lacZ* fusion in the ∆*folE_IB1* background (Table S3). Absence of one FolE resulted in up-regulation of the expression of the gene for the other one, which indicated regulation of expression of both genes by folate, THF, or a product of fTHF, such as S-adenosyl-methionine in *E. coli* (41, 42) but not by ZMP or ZTP.

### Influence of different growth media

Following single ∆*folE* deletions in all three genes, two marker-free double mutants were also constructed; however, the ∆*folE_IA* ∆*folE_IB1* mutant was made with a disruption in the *folE_IB1* gene instead of as a marker-free deletion. It was not possible to construct a triple mutant; at least one FolE enzyme seems to be essential in *C. metallidurans. C. metallidurans* strain AE104 and its cognate ∆*folE* mutants were cultivated in different Tris-buffered mineral salt media, medium zinc (M1, mZn), low zinc (M1a, lZn), low magnesium (M1b, lMg), low zinc and low magnesium or low metal (M2, lZnMg, lM), and low iron (M3, lFe) TMM to study the influence of the respective metal supply on the growth of these strains.

The metal content of these strains was determined using ICP-MS (Table 5; Table S4). No *folE* deletion had an effect on the cellular metal content. There was no cross-talk between missing FolEs and metal homeostasis, with the exception of the Zur-dependent control of *folE_IB2* expression. The magnesium, iron, nickel, and manganese contents of the cells cultivated in the media with sufficient iron were unchanged. The zinc content decreased by about 50% (from about 70,000 Zn/cell to 20,000 to 30,000), and the cobalt content decreased even more (from about 25,000 to below 1,000 Co/cell) in low zinc and low metal but not in low magnesium TMM. These findings indicate that the trace element solution SL6 was an important source of zinc and cobalt. Cells cultivated in low zinc or low metal media were zinc and cobalt deprived but not those grown in medium zinc or low magnesium TMM (Table 5). The different Mg concentrations in medium zinc compared to low magnesium, as well as in low zinc compared to low metal TMM, did not influence the metal composition of the cells.

**TABLE 5:** Metal content of *C. metallidurans* strain AE104 and mutants with deletions in the genes for FolE_I-type enzymes^*a*^

Strain	Medium	Mg × 10^6^	Fe × 10^4^	Zn × 10^3^	Co × 10^2^	Ni × 10^2^	Mn × 10^2^
AE104	M1 mZn	12.7 ± 2.3	85.9 ± 19.5	68.8 ± 17.3	268 ± 72	23.6 ± 8.46	3.94 ± 2.13
	M1a lZn	12.8 ± 1.3	93.1 ± 12.2	**33.1 ± 7.4**	**7.01 ± 1.73**	25.7 ± 8.48	3.84 ± 1.63
	M1b lMg	12.6 ± 1.9	99.2 ± 17.3	80.4 ± 19.1	253 ± 29	33.3 ± 9.63	8.54 ± 2.58
	M2 lM	14.3 ± 0.74	103 ± 8.5	**26.5 ± 10.8**	**6.07 ± 1.14**	33.3 ± 9.63	3.98 ± 0.94
∆*folE_IA*	M1 mZn	12.6 ± 1.6	85.5 ± 13.3	71.5 ± 18.5	204 ± 74	15.5 ± 5.28	6.98 ± 5.03
	M1a lZn	12.6 ± 1.1	93.7 ± 7.1	**19.1 ± 3.6**	**6.29 ± 1.26**	26.1 ± 7.28	3.83 ± 1.03
	M1b lMg	15.2 ± 1.6	111 ± 10.4	80.4 ± 5.6	296 ± 116	14.8 ± 0.08^*b*^	14.0 ± 3.80^*c*^
	M2 lM	13.2 ± 2.40	105 ± 12.3	**25.2 ± 7.95**	**12.6 ± 1.78**	40.3 ± 10.2	3.94 ± 0.26
∆*folE_IB1*	M1 mZn	14.1 ± 4.3	81.7 ± 16.9	78.0 ± 13.7	243 ± 85	19.7 ± 5.68	6.40 ± 3.46
	M1a lZn	12.7 ± 1.5	92.7 ± 12.0	**20.7 ± 2.7**	**7.60 ± 1.17**	21.9 ± 2.50	4.22 ± 1.87
	M1b lMg	11.3 ± 1,2	77.9 ± 8.27	59.4 ± 18.0	188 ± 46	9.99 ± 1.69	3.92 ± 1.77
	M2 lM	12.9 ± 1.13	98.2 ± 6.27	**26.3 ± 6.11**	**6.22 ± 0.90**	38.1 ± 9.04	3.70 ± 0.29
∆*folE_IB2*	M1 mZn	13.3 ± 3.0	83.6 ± 18.3	84.3 ± 14.6	262 ± 70	19.4 ± 3.44	4.61 ± 2.00
	M1a lZn	14.0 ± 0.9	97.4 ± 9.6	**22.6 ± 5.9**	**8.79 ± 2.14**	28.1 ± 11.2	3.63 ± 0.89
	M1b lMg	13.3 ± 1.1	97.9 ± 14.2	61.2 ± 4.0	289 ± 64	24.9 ± 0.03^*b*^	3.15 ± 1.06
	M2 lM	12.5 ± 1.05	101 ± 10.8	**26.5 ± 6.11**	**6.50 ± 1.57**	42.6 ± 16.5	5.76 ± 1.76
∆*folE_IA ∆folE_IB2*	M1 mZn	13.6 ± 1.2	92.8 ± 10.9	49.4 ± 2.4	303 ± 34	16.6 ± 3.05	1.63 ± 0.30^*b*^
∆*folE_IB1 ∆folE_IB2*	M1 mZn	13.8 ± 0.8	87.4 ± 3.3	73.3 ± 13.8	202 ± 98	12.3 ± 3.10	1.33 ± 0.01^*b*^
∆*folE_IA ∆folE_IB1*	M1 mZn	14.5 ± 0.7	117 ± 6.6	55.9 ± 5.2	460 ± 44	29.1 ± 8.65	2.04 ± 0.12^*b*^

^*a*^ The metal content in atoms per cell was determined with the ICP-MS of *C. metallidurans* strain AE104 and its ∆*folE_IA, ∆folE_IB1,* and ∆*folE_IB2* mutants in Tris-buffered mineral salts medium M1 (mZn), M1a (lZn, no SL6), M1b [lMg, 0.1 mM Mg(II)] or M2 [lM, no SL6, and 0.1 mM Mg(II)]. Bold, difference to the AE104 value in M1 with D >1. Three biological repeats, deviations indicated; n.a., not analyzed.

^*b*^ Only one result, reproductions were below detection limit, technical deviation given.

^*c*^ Mean value of two results, reproduction below detection limit and therefore not different from the parent value.

**Fig 2:**
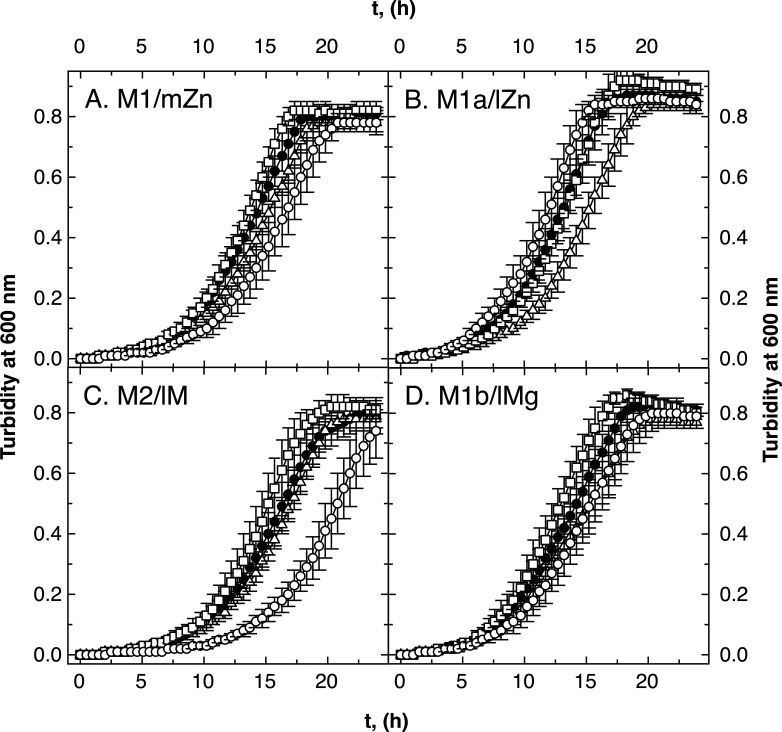
Growth of ∆*folE_I* mutants of strain AE104 in various media. Growth of the *C. metallidurans* strains in medium zinc TMM (panel A, M1/mZn), low zinc TMM (no SL6, panel B, M1a/lZn), low zinc and magnesium [M2/lM, no SL6, and 0.1 mM Mg(II), panel C], and low magnesium [M1b/lMg, 0.1 mM Mg(II), panel D] in 96-well plates is shown. Strains were AE104 parent (closed circles, ●), ∆*folE_IB2* (open triangles, ∆), ∆*folE_IB1* (open squares, □ ), and ∆*folE_IA* (open circles, ◯). *N* >3, deviations shown.

In growth experiments (Fig. 2), no difference between medium zinc- and low magnesium-grown cells was visible in the AE104 background. Although the cellular zinc and cobalt content of low zinc and low metal cells were in a comparably low range (Table 5), the absence of FolE_IB2, which was only produced under zinc starvation conditions [Fig. S1 (5, 17)], affected growth in low zinc but not in the low metal medium (Fig. 2B, open triangles). In contrast, the absence of FolE_IA influenced growth in low metal but not in low zinc (Fig. 2C, open circles). Although a lower magnesium concentration in the growth medium (100 µM compared to 1 mM) did not change the metal composition of the cells (Table 5), it altered the importance of the individual FolEs for growth under cobalt and zinc starvation conditions. High Mg plus low Co/Zn conditions required FolE_IB2, while low Mg plus low Co/Zn increased reliance on FolE_IA.

The growth rates of the single- and double-mutant strains in the AE104 background were calculated (Table S5). Strain AE104 grew with an approximately 10% higher growth rate in low magnesium and a 10% lower rate in low metal TMM. The *folE* gene deletions did not change the growth rates, so that the delayed growth of the ∆*folE_IB2* mutant in low zinc and of the ∆*folE_IA* mutant in low metal was the result of an extended lag phase. Sufficient amounts of THF were important to initiate the exponential phase of growth.

In low iron without added iron and trace element solution SL6, the cellular iron, magnesium, zinc, and cobalt contents of parent AE104 and its ∆*folE* mutants were decreased, while the number of Mn atoms was increased (Table S4); however, there were no differences between parent and mutants. Growth of all strains was delayed (Fig. S6) with no difference noticeable between parent AE104 and its cognate ∆*folE* mutants. The higher Mn content of the cells was insufficient to substitute for the missing iron, cobalt, or zinc ions. The cells suffered more from general iron starvation than from insufficient activation of the FolE_IBs.

**Fig 3:**
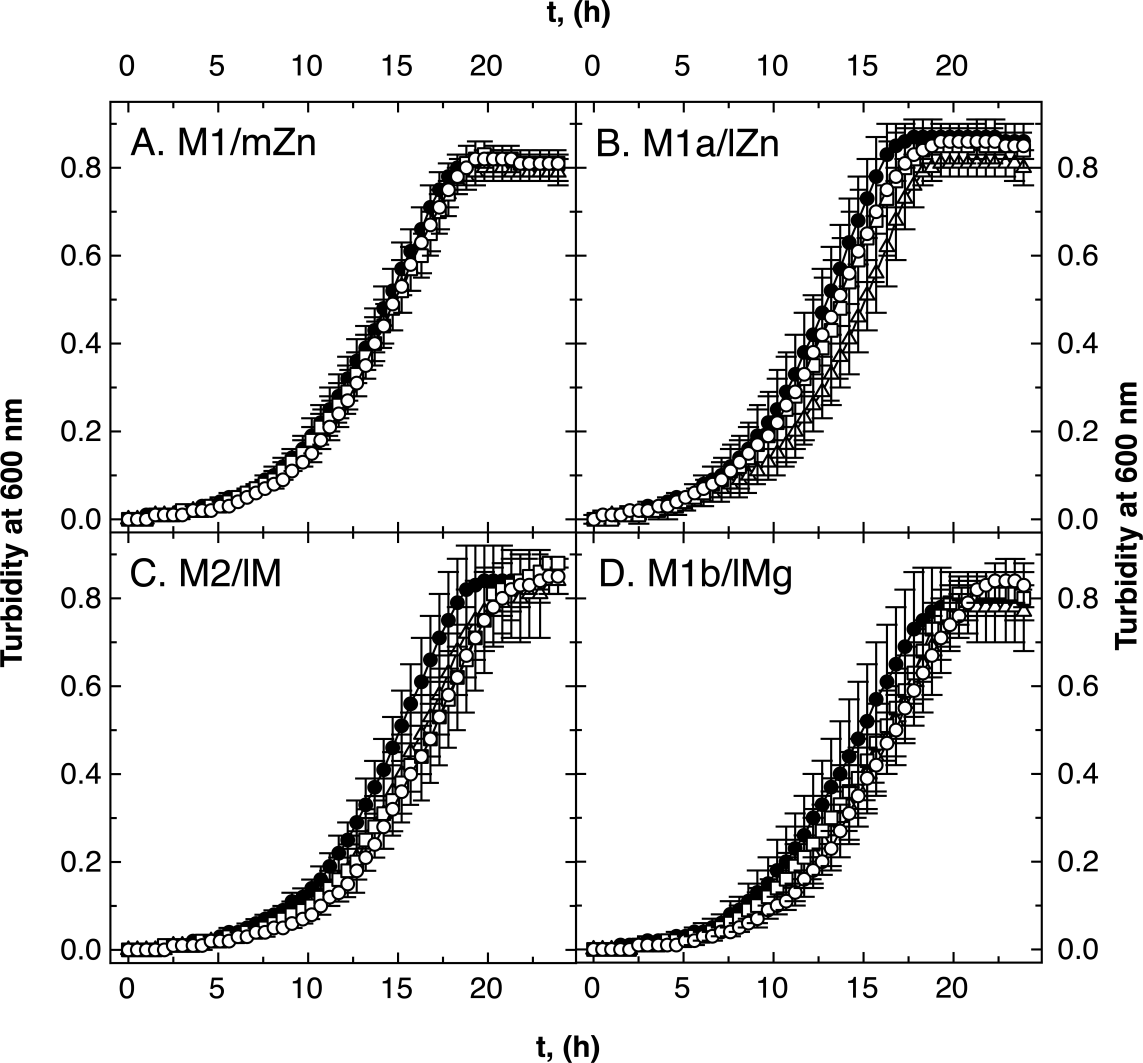
Growth of ∆*folE_I* double mutants in various media. Growth of the *C. metallidurans* in medium zinc TMM (panel A, M1/mZn), low zinc TMM (no SL6, panel B, M1a/lZn), low zinc and magnesium [M2/lM, no SL6, and 0.1 mM Mg(II), panel C], and low magnesium [M1b/lMg, 0.1 mM Mg(II), panel D] in 96-well plates is shown. Strains were AE104 parent (closed circles, ●), ∆*folE_IB2* ∆*folE_IA* meaning B1 only (open triangles, ∆), ∆*folE_IB2* ∆*folE_IB1* or A only (open squares, □), ∆*folE_IA* ∆*folE_IB1::disrupted* or B2 only (open circles, ◯), *N* >3, deviations shown.

There was no difference between growth of the three double-null *folE* mutants and the parental strain AE104 in the medium zinc, low zinc, low magnesium, or low metal media (Fig. 3). Deletion of a second *folE* gene compensated for the dependence on FolE_IB2 in low zinc medium and vice versa that of FolE_IA in low metal medium. In the low zinc and low metal media, two FolEs could not be sufficiently produced or activated to allow wild-type growth, which highlights the importance of the respective third FolE. The deletion of a second FolE ameliorated this effect.

In low iron medium, essentially iron, zinc, and cobalt starvation medium, the ∆*folE_IA ∆folE_IB1::disrupted* strain, which contained only FolE_IB2, grew more slowly (Fig. S6D). However, this strain grew similarly to the parental strain in the other growth media, so that FolE_IB2 alone was able to initiate production of sufficient amounts of THF in these media, but when additionally iron was low.

To analyze whether this effect was due to a limited allocation of iron to FolE_IB2, the experiment was repeated with low iron medium supplied with 0.5-µM iron ammonium citrate (Fig. S7). All three double mutants grew more slowly compared to the parent: the strain with only FolE_IA grew without a significant difference to the parent (open squares); the strain with only FolE_IB1 grew with a small difference (open triangles); and the strain with only FolE_IB2 grew with a large difference (open circles). Poorer growth of the FolE_IB2-only double mutant in M3 medium could not be compensated by supplying additional iron.

At least one FolE was essential for *C. metallidurans*. The difference between the single and double mutants indicated that FolE_IA was required under Co/Zn starvation and low magnesium conditions due to some negative interference between the two FolE_IBs. FolE_IB2 was important under Co/Zn starvation and high magnesium availability. When the iron content of the medium was additionally decreased, FolE_IB1 and FolE_IA could be sufficiently activated in double mutants containing only this particular FolE but not FolE_IB2. Added iron did not compensate this effect.

### Influence of the metal-handling ability of the cells

The zinc uptake and distribution pathway should be changed in ∆*zupT* mutants of strain AE104, which is impaired in zinc allocation to the RNA polymerase subunit RpoC due to the absence of this zinc importer. An effect of ∆*zupT* should be reversed in ∆*zur* mutants, which strongly up-regulated expression of the components of the Zur regulon, including *zupT* and operon Op0317f_1 that contains *folE_IB2* (4, 5, 17). Thus, the ∆*zupT* and ∆*zur* mutants served as respective parental strains for the introduction of ∆*folE* deletion mutations.

In the ∆*zupT* strain, the Mg, Fe, Mn, and Ni contents were unchanged in the parental ∆*zupT,* its mutants, ∆*zur* and most of its mutants; however, the magnesium content was increased in ∆*zur* ∆*folE_IA* in medium and in low zinc media M1 and M1a (Table 6). Similar to the AE104 strains, the cobalt atoms per cell were lower in cells grown in media without SL6, namely M1a and M2, and were barely different from the cobalt content of AE104 and its *∆folE* mutants (Table 5). The zinc content of all ∆*zupT* strains was on a similarly low level in all media, as expected due to the absence of zinc uptake systems, and this level was similar to that of strain AE104 cultivated in lZn media M1a and M2. The zinc content of ∆*zur* cells was on the AE104 level in M1-grown cells (mZn) but also decreased in M1a-grown cells (lZn, Table 6). While parent AE104 cells possessed sufficient Zn and Co in medium zinc and low magnesium (being also medium zinc) media, the *zupT* mutant cells were zinc starved in all media and also showed Co starvation but only in low zinc and low metal media (M1a, M2). The ∆*zur* cells contained similar levels of zinc and cobalt as the parental strain, but the components of the Zur regulon were constitutively up-regulated (4, 17).

**TABLE 6:** Metal content of derivatives of *C. metallidurans* ∆*zupT* and ∆*zur* with deletions in the genes for FolE_I-type enzymes^*a*^

Strain	Medium	Mg × 10^6^	Fe × 10^4^	Zn × 10^3^	Co × 10^2^	Ni × 10^2^	Mn × 10^2^
*∆zupT*	M1 mZn	12.3 ± 1.59	91.7 ± 14.4	33.1 ± 12.4	113 ± 28.0	36.7 ± 3.90	1.25 ± 0.30
	M1a lZn	12.6 ± 1.41	92.5 ± 16.2	18.4 ± 4.06	**3.31 ± 0.45**	36.6 ± 6.13	2.67 ± 0.61
	M1b lMg	11.6 ± 0.43	89.5 ± 5.51	48.5 ± 13.1	150 ± 8.16	76.4 ± 2.47	3.07 ± 0.82
	M2 lM	11.5 ± 0.03	97.1 ± 1.8	28.4 ± 5.04	**14.4 ± 0.27**	41.1 ± 0.98	1.89 ± 0.06
*∆zupT ∆folE_IA*	M1 mZn	14.1 ± 2.05	87.4 ± 13.7	36.0 ± 10.3	113 ± 35.3	26.7 ± 12.2	2.93 ± 1.81
	M1a lZn	14.0 ± 1.61	97.3 ± 16.8	18.5 ± 5.80	**3.30 ± 0.21**	10.7 ± 1.80	3.67 ± 0.77
	M1b lMg	13.6 ± 0.43	77.1 ± 29.4	47.3 ± 17.0	121 ± 52.3	24.0 ± 15.7	4.18 ± 2.92
	M2 lM	13.8 ± 2.45	110.1 ± 11.6	23.8 ± 4.51	**11.8 ± 1.36**	21.8 ± 7.43	2.92 ± 0.69
*∆zupT ∆folE_IB1*	M1 mZn	13.9 ± 1.05	93.3 ± 12.4	45.9 ± 3.75	88.2 ± 29.4	21.5 ± 13.1	2.98 ± 1.95
	M1a lZn	14.6 ± 1.20	99.7 ± 10.1	28.6 ± 6.24	**3.35 ± 1.01**	15.7 ± 1.04	3.23 ± 0.44
	M1b lMg	11.9 ± 0.96	75.8 ± 8.97	35.3 ± 4.56	106 ± 19.4	10.4 ± 0.80	2.35 ± 0.77
	M2 lM	14.2 ± 2.64	109.4 ± 26.5	30.0 ± 2.30	**12.8 ± 6.06**	18.3 ± 4.78	3.63 ± 2.19
*∆zupT ∆folE_IB2*	M1 mZn	12.8 ± 1.39	94.5 ± 19.1	34.1 ± 7.31	75.4 ± 13.4	17.3 ± 7.99	1.69 ± 0.54
	M1a lZn	14.0 ± 1.61	97.3 ± 16.8	18.5 ± 5.80	**3.30 ± 0.21**	10.7 ± 1.80	2.47 ± 0.45
	M1b lMg	9.05 ± 0.52	76.0 ± 5.44	36.6 ± 6.70	78.5 ± 4.74	14.9 ± 6.10	3.21 ± 0.40
	M2 lM	13.8 ± 2.45	110.1 ± 11.6	23.8 ± 4.51	**11.8 ± 1.36**	21.8 ± 7.43	3.15 ± 0.46
							
∆*zur*	M1 mZn	12.9 ± 1.84	100 ± 6.87	55.7 ± 12.3	355 ± 49.5	32.7 ± 5.82	1.88 ± 0.19
	M1a lZn	12.6 ± 1.56	96.6 ± 3.73	**18.8 ± 3.51**	**5.82 ± 0.59**	39.5 ± 3.27	1.88 ± 2.50
	M1b lMg	13.8 ± 1.24	105 ± 9.43	71.4 ± 4.24	393 ± 37.2	50.7 ± 11.4	4.50 ± 1.55
*∆zur ∆folE_IA*	M1 mZn	**32.8 ± 4.04**	78.7 ± 4.40	54.4 ± 9.38	314 ± 35.8	32.5 ± 2.88	1.92 ± 0.10
	M1a lZn	**34.3 ± 5.60**	76.1 ± 2.28	**19.7 ± 2.70**	**4.50 ± 1.07**	26.5 ± 4.23	4.15 ± 1.92
	M1b lMg	27.2 ± 4.29	80.7 ± 9.84	69.4 ± 9.41	285 ± 51.7	55.1 ± 4.97	5.61 ± 2.22
*∆zur ∆folE_IB1*	M1 mZn	12.7 ± 0.75	98.9 ± 3.19	54.3 ± 11.4	366 ± 43.1	27.7 ± 3.14	1.89 ± 0.72
	M1a lZn	11.7 ± 1.60	100 ± 10.7	**15.2 ± 2.34**	**4.72 ± 1.55**	46.0 ± 12.1	2.25 ± 0.64
	M1b lMg	14.1 ± 1.12	112 ± 7.27	**104 ± 15.8**	365 ± 4.39	35.1 ± 8.10	n.d.
*∆zur ∆folE_IB2*	M1 mZn	13.2 ± 0.65	101 ± 4.59	62.7 ± 9.41	368 ± 20.1	29.0 ± 4.15	1.58 ± 0.67
	M1a lZn	12.9 ± 0.33	98.2 ± 3.43	**20.4 ± 7.15**	**6.48 ± 0.91**	40.0 ± 2.83	2.50 ± 0.67
	M1b lMg	10.9 ± 1.58	89.4 ± 1.70	64.4 ± 11.8	236 ± 17.1	56.0 ± 35.4	3.04 ± 1.32

^*a*^ The metal content in atoms per cell was determined with the ICP-MS of *C. metallidurans* strains ∆*zupT* and ∆*zur* and its ∆*folE_IA, ∆folE_IB1,* and ∆*folE_IB2* mutants in Tris-buffered mineral salts medium M1 (mZn), M1a (lZn, no SL6), M1b [lMg, 0.1 mM Mg(II)], or M2 [lM, no SL6, 0.1 mM Mg(II)]. Bold, difference to the value of the respective parent strain in M1 with *D* >1. Three biological repeats, deviations indicated; n.d., not detected.

**Fig 4:**
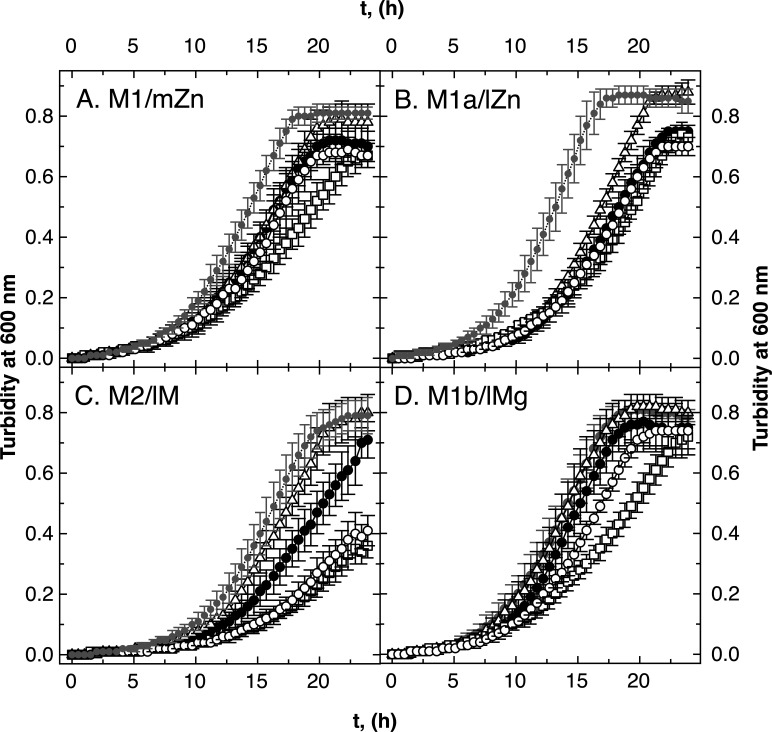
Growth of ∆*zupT ∆folE* mutants. Growth of the *C. metallidurans* strains in medium zinc TMM (panel A, M1/mZn), low zinc TMM (no SL6, panel B, M1a/lZn), low zinc and magnesium [M2/lM, no SL6, and 0.1 mM Mg(II), panel C], and low magnesium [M1b/lMg, 0.1 mM Mg(II), panel D] in 96-well plates is shown. Strains were AE104 parent (closed circles in gray, ●), ∆*zupT* (closed circles, ●), ∆*zupT* ∆*folE_IB2* (open triangles, ∆), ∆*zupT* ∆*folE_IB1* (open squares, □), and ∆*zupT* ∆*folE_IA* (open circles, ◯). *N* >3, deviations shown.

**Fig 5:**
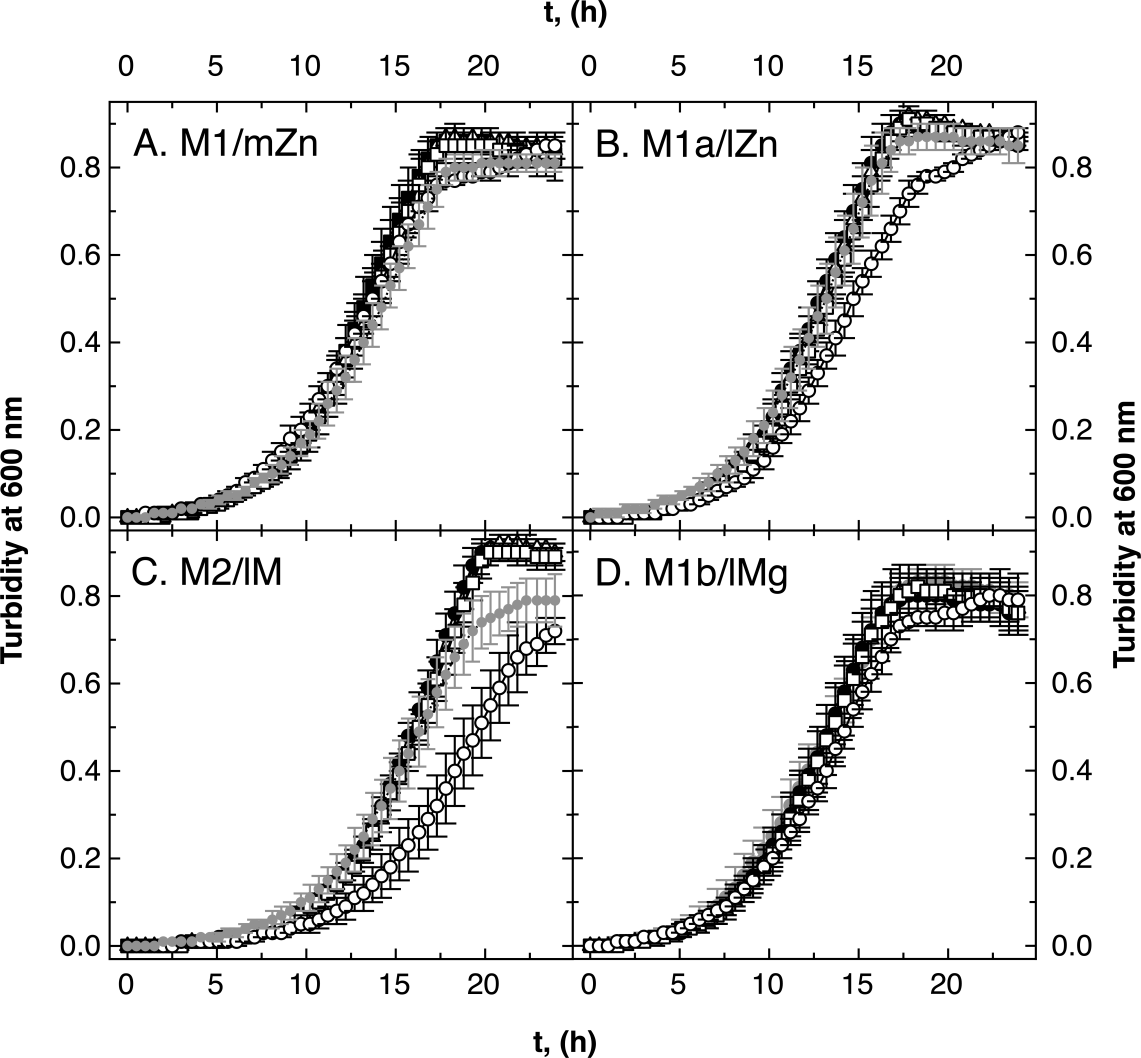
Growth of ∆*zur ∆folE_IA* mutants. Growth of the *C. metallidurans* strains in medium zinc TMM (panel A, M1/mZn), low zinc TMM (no SL6, panel B, M1a/lZn), low zinc and magnesium [M2/lM, no SL6, and 0.1 mM Mg(II), panel C], and low magnesium [M1b/lMg, 0.1 mM Mg(II), panel D] in 96-well plates is shown. Strains were AE104 parent (closed circles in gray, ●), ∆*zur* (closed circles, ●), ∆*zur* ∆*folE_IB2* (open triangles, ∆), ∆*zur* ∆*folE_IB1* (open squares, □), and ∆*zur* ∆*folE_IA* (open circles, ◯). *N* >3, deviations shown.

All ∆*zupT* strains grew more slowly compared to strain AE104 in the four media (M1–M2, Fig. 4), but the ∆*zur* strains did not (Fig. 5). The decreased cellular zinc content in ∆*zupT* cells in media M1 and M1b compared to AE104 cells resulted in an increased importance of FolE_IB1 in these cells (Fig. 4A and D, open squares). As the growth rates were unaffected (Table S5), the ∆*zupT* cells had problems to provide sufficient zinc to FolE_IA during the lag phase. Such an effect was not observed in ∆*zur* cells (Fig. 5) nor in the parental background (Fig. 2). In analogy with what has been shown for RpoC (13), ZupT was required to provide sufficient zinc to zinc-dependent FolE_IA.

In low zinc and cobalt medium (M1a, Fig. 4B), all *∆zupT* strains showed more strongly delayed growth compared to the parental strain AE104 than in other media (Fig. 4). The growth rates were similar between all strains (Table S5), so that the lag phases were affected. In all media, the ∆*zupT ∆folE_IB2* mutant grew better than the ∆*zupT* parent, especially in low zinc medium M1a and in low metal medium M2 (Fig. 4B and C, open triangles). Again, this effect was absent in the cognate *∆zur* strains (Fig. 5). Expression of *folE_IB2* under zinc starvation conditions limited activation of the other FolEs under Co/Zn limitation in the ∆*zupT* mutant.

In low metal medium, the ∆*zupT* ∆*folE_IB1* and ∆*zupT* ∆*folE_IA* mutants grew after an initial delay (M2, Fig. 4C), while the ∆*folE_IB1* mutant grew slightly better than its parent, AE104, but the ∆*folE_IA* mutant was also delayed in growth initiation. All strains grew with similar growth rates, so that again all these observed effects were related to the lag phase (Table S5). As observed for the strains in the AE104 genetic background (Fig. 2), FolE_IA was required for full growth in metal starvation medium M2 in the ∆*zur* background (Fig. 5C).

There was at least one condition that required a particular FolE enzyme. The zinc-dependent FolE_IA was needed in strains AE104 and ∆*zur* in the low metal medium (M2), and even more so for the ∆*zupT* mutant in this medium, but in the *∆zur* mutant, this was only observed in low zinc medium (M1a). These media were also cobalt starvation media, and this metal might not have been sufficiently allocated to the FolE_IBs. Metal-promiscuous FolE_IB1 was required when, under conditions of zinc availability, the metal could not be sufficiently imported and allocated in the absence of ZupT. FolE_IB2 was required when cells were under zinc and cobalt starvation conditions in low zinc but high magnesium medium (M1a). The respective metal cofactor could not be sufficiently allocated to FolE_IA and FolE_IB1, respectively. This might indicate the presence of a common zinc and cobalt allocation pathway or a cross-over of different zinc and cobalt allocation pathways at some point.

### Influence of metal chelators

**Fig 6:**
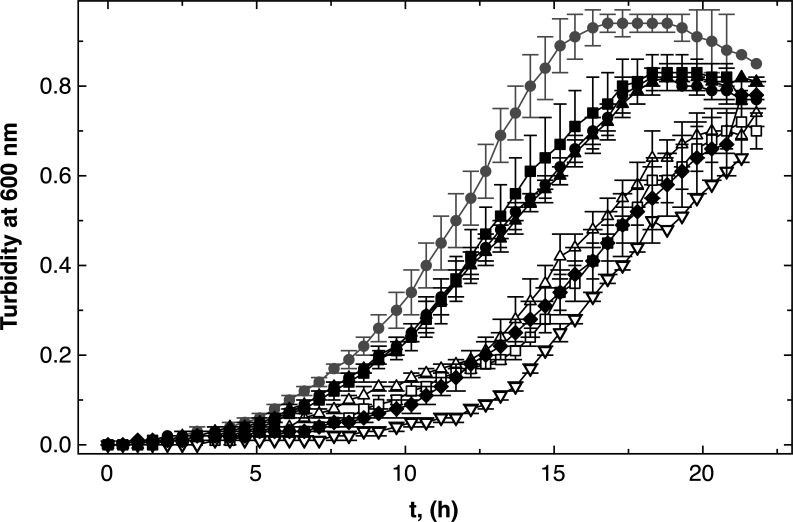
Growth of *∆folE* mutants in the presence of 0.1 mM EDTA. Growth of the *C. metallidurans* strains in medium zinc TMM containing 0.1 mM EDTA in 96-well plates is shown. Strains were AE104 parent without EDTA (closed gray circles, ●), and in presence of 0.1 mM EDTA, the strains AE104 parent (closed circles, ●), ∆*folEI_B1* (closed squares, ■), *∆folE_IA ∆folE_IB1::disrupted* (open inverted triangles,▽)*,* ∆*folE_IB2* ∆*folE_IA* (open, ∆), ∆*folE_IB2* ∆*folE_IB1* (open squares, □), ∆*folE_IA* (closed diamonds, ◆), and ∆*folE_IB2* (closed triangles, ▲). *N* >3, deviations shown.

In the presence of 0.1 mM of the general chelator of transition metal cations EDTA, all strains grew slower than the parental strain AE104 without EDTA (Fig. 6). The ∆*folE_IB1* and ∆*folE_IB2* single-deletion mutants grew similarly to the parent, AE104, in EDTA, but all other mutants showed delayed growth compared to these three. The ∆*folE_IA* mutant displayed the slowest growth of the single mutants, which was comparable to that of the ∆*folE_IB1 ∆folE_IB2* (FolE_IA-only) and ∆*folE_IA ∆folE_IB2* (FolE_IB1-only) double mutants. The double mutant ∆*folE_IA* ∆*folE_IB1::dis* showed a longer lag phase than all the other strains. In the presence of EDTA, FolE_IA was the most important FolE-type enzyme, and its absence could not be compensated by FolE_IB1 and FolE_IB2. EDTA inhibited metalation of the promiscuous FolE_IBs more than that of FolE_IA.

The metal content of the AE104 derivatives did not change in the presence of EDTA with the exception of a lower cobalt content (Table S6). When the gene for the zinc importer ZupT was deleted, the cellular zinc and cobalt content of the cells was decreased, but EDTA did not decrease these numbers further (Table S6). The growth rates of the ∆*folE* single mutants of parent AE104 and parent ∆*zupT* were not significantly different (Table S7) despite a lower growth rate of all ∆*zupT* strains compared to those with ZupT. All growth effects were again the result of a changed lag phase.

EDTA, membrane-permeant TPEN, and DIP complex divalent transition metal cations with similar stability constants, EDTA with Cu > Ni > Zn > Co > Fe = Mn, TPEN with a similar ranking but higher stability constants, and DIP with Cu = Fe > Zn in an inverted arrangement (43–45). The *∆folE* single and double mutants grew with only a slight delay compared to the parental strain AE104 in the presence of TPEN (Fig. S8), and the IC_50_ values for the ∆*folE* single mutants and TPEN were not different from that of the parent AE104 (Table S8). When the gene for the zinc importer ZupT was deleted, the IC_50_ values of all strains decreased below 0.5 µM TPEN, again without differences between the mutants and the ∆*zupT* parent (Fig. S8C). Deletion of the *cobW1* gene for a possible zinc-delivering GTPase did not change the growth of the *cobW1* mutant and its ∆*folE* derivatives compared to strain AE104 and its respective mutants (Fig. S8D). Due to large deviations, not much influence of TPEN on the growth of the ∆*folE* mutants could be measured. The different effects of EDTA and membrane-permeable TPEN indicated that the limited external availability of the metal ions was responsible for the decreased activation of the metal-promiscuous FolE_IBs in EDTA-grown cells. In contrast, TPEN mediated an overall decrease of metal availability.

In the presence of DIP, growth of both ∆*folE_IB* mutants of strain AE104 was similar to their parent, but the ∆*folE_IA* mutant was more sensitive to DIP (Fig. S9A) with a decrease in the IC_50_ value from about 60 µM to 36 ± 7 µM (Table S8). The zinc content of the four strains in the presence of 25 µM DIP was similar, but the cobalt content was decreased (Table S6). Deletion of *zupT* decreased DIP resistance, but there was no longer a difference between ∆*zupT* and its three ∆*folE* single mutants (Fig. S9B). The ∆*folE_IA ∆folE_IB1::dis* mutant, but not the other two double mutants, displayed a decreased DIP resistance (Fig. S10).

Despite the differences in the metal affinities between DIP and EDTA, resistance to either chelator was not influenced by a ∆*folE_IB1* or ∆*folE_IB2* mutation, but it was affected by a ∆*folE_IA* single-deletion mutation. Clearly, zinc could still be efficiently allocated to FolE_IA. Growth in the presence of membrane-permeant TPEN, or deletion of the *zupT* gene, removed the differences between the ∆*folE* deletion mutants and their parent. Controlled uptake and cytoplasmic allocation of metals were important to activate the promiscuous FolE_IBs. In the presence of DIP, the FolE_IA-only and FolE_IB1-only mutants grew better than the AE104 parental strain and, again, the FolE_IB2-only double mutant displayed a delayed growth phenotype (Fig. S10), which may be the result of a decreased iron availability.

### Inhibitors of THF synthesis

**Fig 7:**
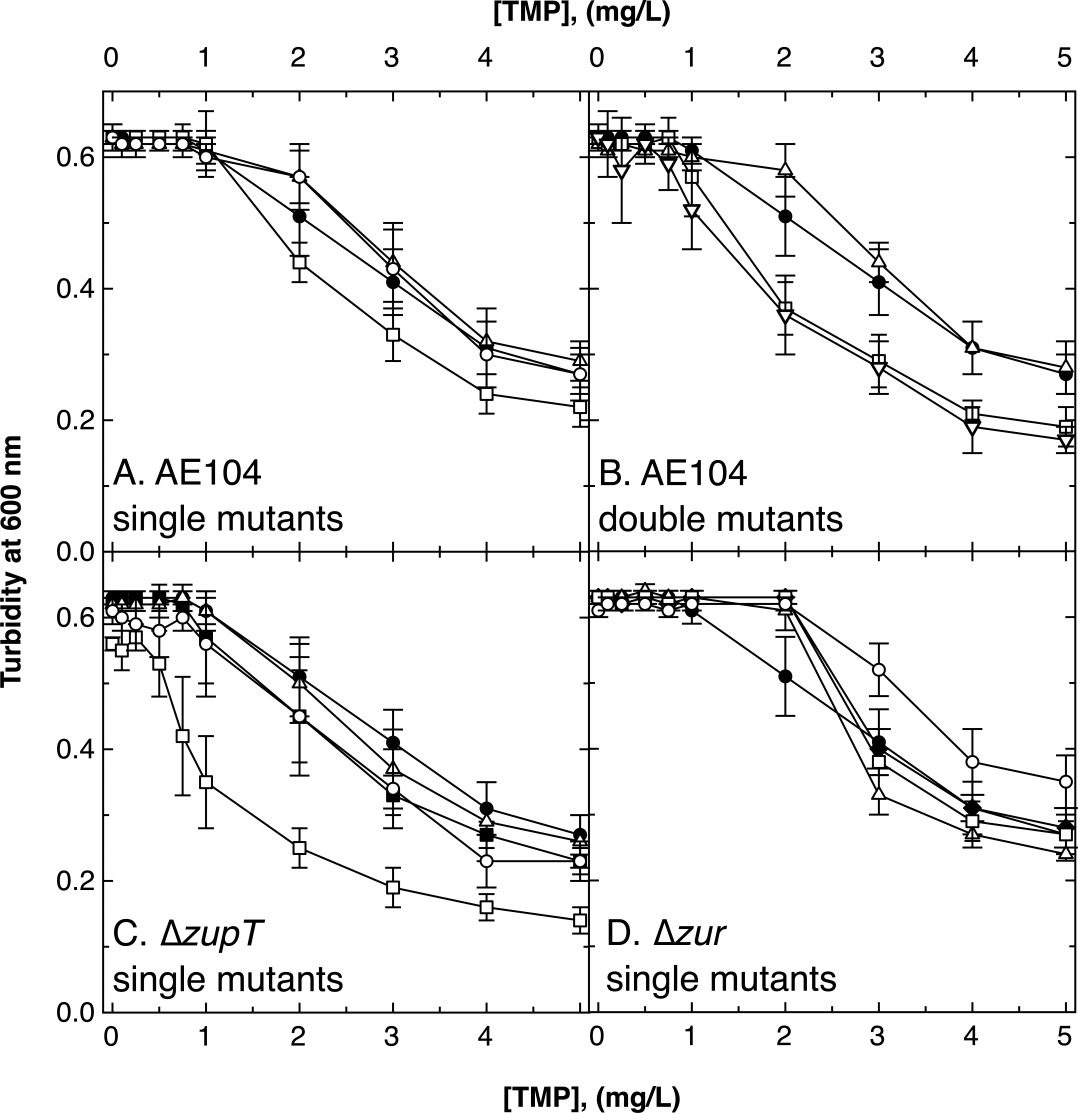
Growth of *∆folE* mutants in the presence of trimethoprim. Final turbidity of *C. metallidurans* strains in medium zinc TMM containing varying concentrations of trimethoprim is shown for derivatives of strain AE104 single (panel A) and double (panel B) mutants, ∆*zupT* (panel C), and ∆*zur* single mutants (panel D). For the single mutants, strains are AE104 parent (closed circles, ●) shown in all panels, ∆*zupT* (closed squares, ■), ∆*zur* (closed diamonds, ◆), and their respective single mutants ∆*folE_IB2* (open triangles, ∆), ∆*folE_IB1* (open squares, □), and ∆*folE_IA* (open circles, ◯). In panel B, the double mutants were *∆folE_IA ∆folE_IB1::disrupted* (open inverted triangles, ▽), ∆*folE_IB2* ∆*folE_IA* (open triangles, ∆), and ∆*folE_IB2* ∆*folE_IB1* (open squares, □). *N* >3, deviations shown.

Trimethoprim and sulfonamide interfere with THF biosynthesis in steps downstream of the reaction catalyzed by the FolE_I-type enzymes. Neither substance induced an up-regulation of *folE_IB1* or *folE_IA* (Table S2), but TMP did induce expression of *pfl-glyA* (Table S1). The ∆*folE_IB1* mutant of strain AE104 was less resistant to trimethoprim than its wild-type parent or the other two ∆*folE* deletion mutants (Fig. 7A), and the IC_50_ for trimethoprim decreased from 4 to 3.3 mg/L (Table S9). Resistance to trimethoprim was not affected in the ∆*folE_IB2 ∆folE_IA* mutant with FolE_IB1 only, but it was in the two double mutants that carried a ∆*folE_IB1* deletion (Fig. 7). Resistance to TMP decreased in the ∆*zupT ∆folE_IB1* but not in the ∆*zur ∆folE_IB1* mutant (Fig. 7B and C). Trimethoprim-mediated stress revealed the importance of FolE_IB1 as well as ZupT-mediated zinc delivery to FolE_IA. Since TMP decreased the concentration of fTHF as shown by the up-regulation of *pfl-glyA-lacZ* expression, an increased folate production was needed to counteract inhibition of a later step of folate biosynthesis by TMP. This explains the need for the activity of both FolE_IA and FolE_IB1 to resist TMP inhibition. Despite a lower specific *in vivo* activity of FolE_IB1 compared to FolE_IA, FolE_IB1 proved more important for this resistance to TMP.

**Fig 8:**
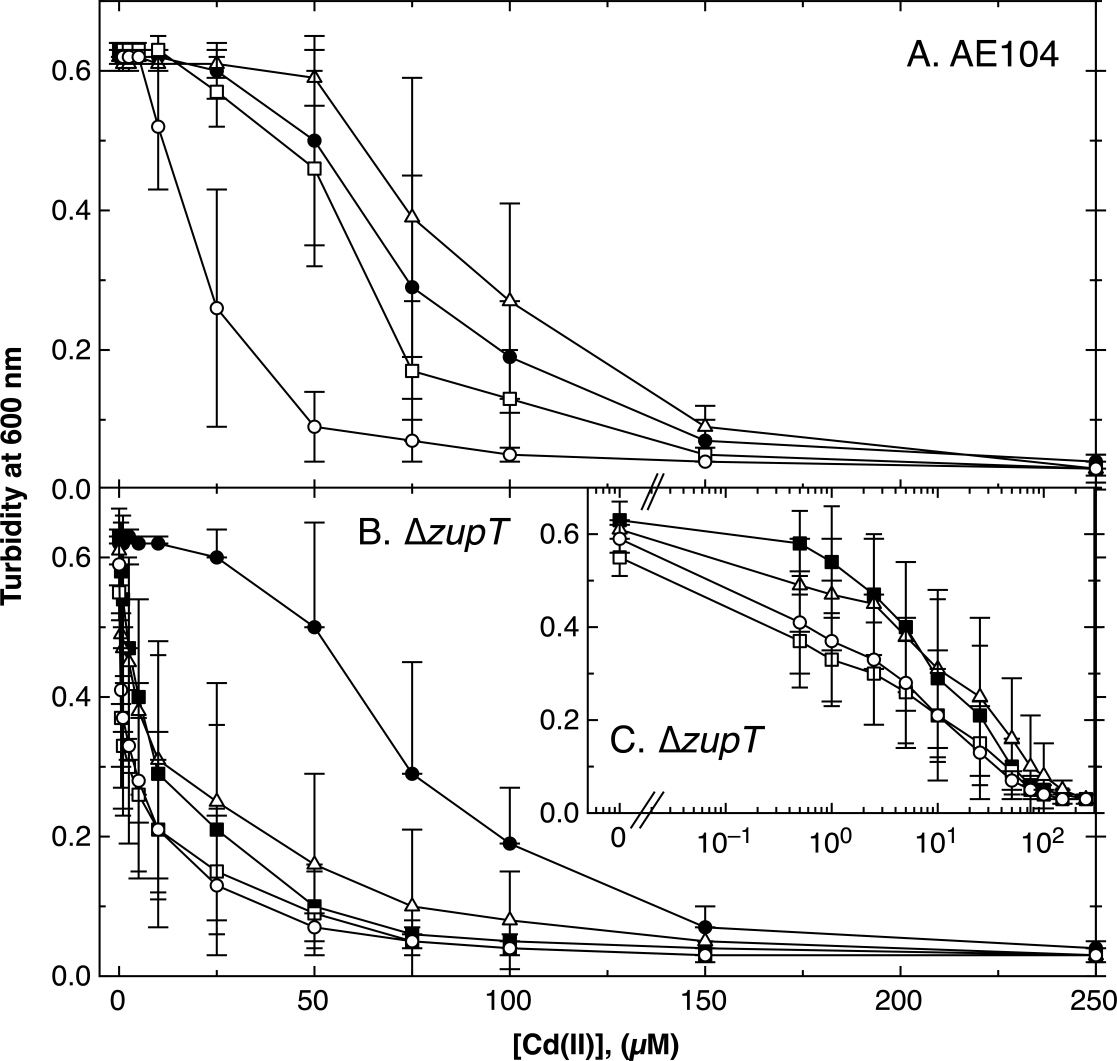
Comparison of cadmium resistance of ∆*folE* single mutants in the strain AE104 and ∆*zupT* background. Final turbidity of *C. metallidurans* strains in medium zinc TMM containing varying concentrations of Cd(II) after 20 h (AE104) or 24 h (∆*zupT* due to the slower growth) is shown for derivatives of strain AE104 (panel A) and ∆*zupT* single mutants (panel B; panel C shows a magnification for low cadmium concentrations). Strains are AE104 parent (closed circles, ●) shown in panels A and B, ∆*zupT* (closed squares, ■), and their respective single mutants ∆*folE_IB2* (open triangles, ∆), ∆*folE_IB1* (open squares, □), and ∆*folE_IA* (open circles, ◯).

No *folE* single deletion altered resistance to sulfonamide in strain AE104 (Fig. S11, Table S8), but the double deletions ∆*folE_IB2* ∆*folE_IB1* (or only FolE_IA present) and ∆*folE_IB2* ∆*folE_IA* (or only FolE_IB1 present) resulted in a decrease of sulfonamide resistance by 50% (Table S9, Fig. S11B). In contrast to TMP, SUAM resistance of the ∆*folE_IA ∆folE_IB1::dis* double mutant was similar to the parent strain (Fig. 8). The effect of an additional ∆*zupT* deletion mutation on the requirement of the *folE* genes was less prominent in the presence of SUAM compared to TMP.

In particular, FolE_IB1 and zinc delivery by ZupT to FolE_IA were required when the last step of THF synthesis was inhibited by TMP. SUAM had a smaller effect on the cellular availability of fTHF as measured by the *plf-glyA-lacZ* reporter but clearly inhibited cells with *folE* deletions. When the rate of the initial step of THF biosynthesis was decreased in these mutants, the decelerated substrate flow through this pathway was no longer able to overcome inhibition of the last synthesis step by SUAM. In general, this can be taken as evidence that the FolEs in *C. metallidurans* were indeed required for the first step of THF biosynthesis *in vivo*. Their full activity was needed when one of the last two steps of this pathway was inhibited.

### Cadmium and oxidative stress

FolE_IA showed *in vitro* a more stable enzyme activity in the presence of added cadmium and hydrogen peroxide than FolE_IB1 (see Table 4). It was tested whether this was also true *in vivo*. The ∆*folE_IA* mutation introduced into strain AE104 clearly resulted in less resistance to cadmium than was shown by the parental strain or, for that matter, by the other two ∆*folE* mutants (Fig. 8A). The ∆*folE_IA* mutant displayed an IC_50_ of 12 µM Cd(II) compared to about 80 µM for the other strains, while cobalt resistance dropped by the half to 81 µM (Table S10). Moreover, this mutant was also less resistant to paraquat and hydrogen peroxide. As expected, the cadmium content increased in AE104 and its ∆*folE* mutant when they were incubated in the presence of 1 µM Cd(II) (Table S11) despite the presence of the cadmium efflux pumps ZntA and CadA under these conditions (3, 46). The cobalt content of parent AE104 and its mutant was strongly decreased, so that cadmium inhibited accumulation of cobalt.

The ∆*zupT* strain was less resistant to cadmium than the parental strain AE104 (Fig. 8) with a strongly decreased IC_50_ (Table S10), while cadmium resistance was even increased in the ∆*zur* mutant. The ∆*zupT ∆folE_IB1* double mutant displayed a decreased IC_50_ for cadmium compared to its parent ∆*zupT* (Table S10), which was similar to that of the ∆*zupT* ∆*folE_IA* strain (Fig. 8C). The ∆*zupT* strain and its mutants contained a higher number of cadmium atoms compared to the AE104 strains and a similar number of cobalt atoms (Table S11), indicating altered metal uptake routes in the ∆*zupT* mutant.

Overall, FolE_IA provided resistance to cadmium, paraquat, and hydrogen peroxide *in vivo* and *in vitro* in strain background AE104. In a ∆*zupT*, but not a ∆*zur*, genetic background, FolE_IA also mediated paraquat and hydrogen peroxide, but not cadmium, resistance. Zinc allocation initiated by ZupT-dependent zinc uptake was necessary for FolE_IA-mediated cadmium resistance. Increased expression of the products of the Zur regulon increased resistance to cadmium and paraquat, and no further effect of a *folE* deletion was evident (Table S10).

## DISCUSSION

### FolEs as the Archilles heel of cellular biochemistry

GTP is not only needed for transcription and translation but also as precursor of important biochemical cofactors. Biosynthesis of queuosine and THF requires as initial step GTP hydrolysis by FolE-type I cyclohydrolases to 7,8-dihydroneopterin triphosphate and that of riboflavin by RibA-type II cyclohydrolases to 2,5-diamino-6-(1-D-ribosylamino)pyrimidin-4(3H)-one-5*'*-phosphate. Moreover, the purine synthesis pathway also provides the precursor of thiamin pyrophosphate, which branches off from phosphoribosylaminoimidazole in a step catalyzed by the zinc-dependent enzyme ThiC (28, 31, 47).

THF synthesis is outstanding compared to the three metabolic routes to thiamin, riboflavin, and queuosine because only GTP synthesis via IMP and GMP specifically requires THF, which is also a cofactor that originates from GTP, so that a feedback loop between THF and GTP exists; no THF is made without GTP and no GTP without THF (Fig. 1). This makes the FolE-dependent GTP cyclohydrolase activity an Achilles heel of the cellular metabolism. The sulfonamides as inhibitor of THF action were consequently the first antagonists of bacterial infection (48).

All the enzymes for purine and THF biosynthesis were identified and quantified in strain AE104, as were those involved in C1-transfer to THF from serine or glycine, serine biosynthesis, inter-conversion of the different C1-THF compounds, and those for important THF-dependent reactions beside purine biosynthesis; methionine, thymidine, pantothenate biosynthesis, and the formylation of methionine-tRNA for initiation of translation (Table S12). The pathway starts with an ATP-dependent pyrophosphorylation of α-D-ribose-5-phosphate to α-D-5-phosphoribosyl-1-pyrophosphate (PRPP) by Prs and leads to 10 biochemical steps, catalyzed by PurF, D, N, L, M, K, E, C, B, H, to inosine-5′-monophosphate. The PurN and PurH steps depend on *N*^10^-formyl-tetrahydroformate (N^10^-formyl-THF). The PurN-mediated reaction can be substituted by PurT, which uses instead of *N*^10^-formyl-THF formate and ATP as energy source in the phosphoribosylglycinamide formyltransferase reaction (Table S12).

PurH catalyzes the *N*^10^-formyl-THF-dependent conversion of AICAR (5′-phosphoribosyl-4-carboxyamide-6-aminoimidazole, Z-nucleotide monophosphate) to IMP, which is transformed in two steps mediated by GuaB and GuaA to GMP. While synthesis of AICAR is possible without THF as cofactor due to the PurT-mediated reaction and, additionally, as by-product of the *de novo* histidine biosynthesis, further conversion of AICAR to IMP, GMP, and finally to GTP strictly requires THF (30–32). Without THF, AICAR (ZMP) accumulates and is phosphorylated by PRPP to ZTP, which is an alarmone signaling a low THF content and interacts with the COG05203 protein ZagA in *B. subtilis* to deliver more zinc to its FolE_IA (25) (Fig. 1).

In *C. metallidurans*, two genes encoding enzymes of purine and THF biosynthesis or their interconversion pathways are located on the bacterial chromid, *folE_IA* and *serA2*. The remaining genes are on the bacterial chromosome (Table S12). Most of the genes are expressed under the control of the house-keeping sigma factor RpoD exclusively or in addition to other sigma factors. The *folE_IA* gene and some other genes are not under RpoD control (49).

Two of these genes were regulated in *C. metallidurans* EDTA-cultivated compared to zinc-treated cells, expression of *purT* encoding the THF-independent phosphoribosylglycinamide formyltransferase was up-regulated and that of *gcvP* encoding a subunit of the glycine cleavage enzyme complex that produces 5,10-methylene-THF was down-regulated (12). This indicates that EDTA-induced metal starvation may result in a limitation of THF. On the one hand, less glycine needs to be degraded to produce formyl-THF, but on the other hand, a formyl-THF-independent bypass reaction was up-regulated 3.25-fold to shuttle more metabolites toward AICAR [ Table S12, (12)], so that formyl-THF was preferentially saved for AICAR transformation into IMP and other important sinks for THF-bound C1 groups.

A BLASTN-mediated search (35) with the sequence of the *pfl* riboswitch (33) resulted in one single match (100% identity) within the *C. metallidurans* genome. This single *pfl* is located at the very 5'-end of the annotated *glyA* gene (Fig. 1); however, this annotated version starts with a “UUG,” unlikely in a GC-rich bacterium, and similarity with GlyA from *E. coli* begins at amino acid Arg97, so that the true open reading frame probably starts with Met94 at an AUG initiation codon. This would locate *pfl* also in the upstream untranslated region as it is usually the case (33). These data indicate that the *pfl*-mediated activity of ZTP in *C. metallidurans* concerns solely *glyA*, suggesting supply of C1 groups to THF via serine and glycine biosynthesis, followed by glycine cleavage.

In *B. subtilis* (Fig. 1), THF starvation results in (i) AICAR/ZMP accumulation; (ii) PRPP-dependent phosphorylation of ZMP to ZTP; (iii) ZTP-mediated activation of the COG05203 protein ZagA; (iv) increased transfer of zinc to a FolE_IA; and (v) subsequently increased GTP-cyclohydrolyzation to form more THF, linking the THF starvation and zinc starvation responses in this firmicute (25). Up-regulation of *purT* and down-regulation of *gcvP* expression under metal starvation conditions in *C. metallidurans* [Table S12, (12)] indicate that a link between THF and zinc metabolism also exists in this beta-proteobacterium (11, 12). Evidence for an interaction of THF biosynthesis and metal homeostasis in *C. metallidurans* has been obtained in this publication; bacterial metal homeostasis is required to protect the Achilles heel of THF biosynthesis. Nevertheless, the regulatory circuits are different between *B. subtilis* and *C. metallidurans* (Fig. 1).

### The three FolEs of *C. metallidurans*

*C. metallidurans* possesses three FolE-type enzymes. FolE_IA depends on zinc (Table 1). The lack of inhibition caused by EDTA and TPEN indicates that the Zn(II) cannot be easily removed from FolE_IA (Table 2). FolE_IA shows some resistance to cadmium ions and H_2_O_2_ *in vitro* and *in vivo* (Table 4, Fig. 8). On the other hand, activity of FolE_IA depends on efficient zinc allocation to this enzyme (Fig. 4). Its specific activity of 40.8 U/g is equivalent to a turnover number of *k*_cat_ = 0.021 s^−1^, which is between the value of the *E. coli* enzyme of 0.05 s^−1^ (29) and that of *Thermus thermophilus* of 0.0035 s^−1^ (50). Assuming a THF concentration in *C. metallidurans* of 50 µM, which would be similar to the FMN concentration in *E. coli* (51), the cellular volume of *C. metallidurans* 0.57 fL (52), and the growth rate 0.3 h^−1^ (Table S3), about two GTP molecules need to be hydrolyzed per second to provide sufficient THF to the cell. With a copy number of 181 ± 112 FolE_IA per cell (27), this equates to roughly four GTPs being hydrolyzed by FolE_IA at *v*_max_, which is sufficient for growth if enough zinc is delivered to FolE_IA. In the *E. coli* enzyme, the zinc ion is coordinated in a Cys-Cys-His environment plus a water molecule (53), and these amino acids are also conserved in the protein from *C. metallidurans* (Fig. S12). As FolE_IA retains its zinc ion avidly, it may be necessary to deliver this ion to the protein immediately upon translation.

The two FolE_IB-type enzymes of *C. metallidurans* do not depend on zinc and are required under conditions of impaired zinc delivery. Both were isolated as inactive holo-forms containing iron, and the metal could be removed from the proteins by EDTA treatment leading to an apo-form. Crucially, both enzymes could be activated in the presence of various metal ions, indicating that both are metal-promiscuous “hop-on-hop-off” enzymes. The highest specific activities of FolE_IB1 and FolE_IB2 attained *in vitro* in the presence of metal cations indicate that the main metal cofactors of both enzymes were Fe, Mn, and Co *in vitro*, which agree with the findings reported for the *B. subtilis* ortholog (28). Together, the two FolE_IB-type enzymes should be able to fully substitute a missing FolE_IA activity.

The ability of FolE_IB1 to hydrolyze about half of the required two GTPs per second may be sufficient should the activity of FolE_IA be decreased due to a lack of zinc. On the other hand, AE104 grown in the standard TMM (M1) medium contained only 394 Mn per cell (Table 5). This would not be a sufficient Mn concentration or number of Mn ions per cell to activate FolE_IB1 or FolE_IB2. The number of Mn atoms per cell is increased under iron starvation (Table S4) to 2,700 Mn per cell, and these numbers would be sufficient to provide Mn ions to the 512 FolE_IB1 per cell. On the other hand, growth of the cells in M3 medium with added 0.5 µM iron (Fig. S7) did not restore the severe growth defect caused by a *folE_IA* gene deletion. This indicates that manganese may not be the primary choice as cofactor for FolE_IB1 *in vivo*. Moreover, *C. metallidurans* does not use manganese in a superoxide dismutase, has no Mn importer of the NRAPM protein family, and accumulates Mn only at a low level (14, 54).

In contrast to Mn, the number of Co ions in M1-grown AE104 cells was 26,800 atoms per cell (Table 5), which may be sufficient to activate both FolE_IB-type enzymes. But in metal starvation media M1a and M2, the number of Co atoms per cell dropped to 701 and 607 per cell, respectively (Table 5), which would be insufficient to activate the FolE_IBs. Because the double mutants that contain only FolE_IB1 or FolE_IB2 were able to grow in low zinc and cobalt media (M1a and M2), this means that iron could also be a metal cofactor for both enzymes under physiological conditions. This agrees with the fact that both enzymes were isolated as iron-containing proteins (Table 1) and with the strong specific activities of the apo-enzymes after anaerobic reconstitution with iron (Table 3). As both FolE_IB enzymes were inactive as isolated, the iron is likely to have been oxidized during the isolation procedure. Indeed, FolE_IB1 was very sensitive to oxidative stress *in vitro* and *in vivo* (Table 4; Table S8).

Taken together, the activity and cellular number of FolE_IA should be sufficient to hydrolyze enough GTP to produce THF and, subsequently, more GTP but only if zinc ions can be efficiently allocated to this protein. Zinc in the environment is usually accompanied by cadmium (55); however, FolE_IA is also able to function in the presence of Cd. This probably allows *C. metallidurans* to thrive in zinc deserts (7). In other environments that contain only small amounts of zinc, *C. metallidurans* relies on FolE_IB1 and FolE_IB2 under conditions of extreme zinc starvation. Both enzymes may depend on iron *in vivo* or on cobalt ions if iron is required for other cellular processes.

### Negative interferences of the three FolEs hint at the existence of cellular metal allocation pathways

Expression of *folE_IB1* and *folE_IA* was not metal regulated nor was expression increased in the presence of inhibitors of THF biosynthesis, although there was an approximate twofold increase in expression when the other FolE was missing (Tables S2 and S3). In *E. coli,* expression of *folE_IA* is controlled by the repressor MetJ (42) and the small regulatory RNA SgrS (56). BLAST (35) revealed neither a MetJ ortholog in *C. metallidurans* nor a nucleotide sequence similar to *sgrS,* so the regulator of expression of these *folE*s in *C. metallidurans* remains unknown.

Control of gene expression by accumulating ZMP via the *pfl* riboswitch (33) exists in *C. metallidurans* as demonstrated by the trimethoprim-mediated up-regulation of a *pfl-glyA-lacZ* reporter gene (Table 1). TMP does not influence expression of *folE_IA* or *folE_IB1*; therefore, ZMP is not the regulator of the expression of these two genes. In agreement with this, a *pfl* riboswitch was only found upstream of *glyA* but not upstream of any of the *folE* genes. In *C. metallidurans*, ZMP controls the loading of C1 moieties onto THF but not the synthesis of this compound (Fig. 1).

The calculated activity of FolE_IA seemed to be sufficient to hydrolyze enough GTP per second and also that of FolE_IB1 as iron-containing enzyme. In agreement with this, the two double mutants containing only FolE_IA (∆*folE_IB1 ∆folE_IB2*) or only FolE_IB1 (∆*folE_IA ∆folE_IB2*) were able to grow in all the media tested (Fig. 3).

The increased sensitivity of the ∆*zupT ∆folE_IB1* mutant to both substances compared to the respective AE104 single-deletion strain clearly demonstrated the importance of ZupT for zinc delivery to FolE_IA. This delivery was also important for growth of *C. metallidurans* under zinc-replete conditions (Fig. 4). FolE_IA and zinc delivery to this enzyme were also important in the presence of the general metal chelators EDTA and DIP (Fig. 6; Fig. S9), indicating that both FolE_IBs could not be sufficiently activated under these conditions.

There was an unexpected difference between the need for the FolEs in low zinc and low metal media (M1a and M2), which were both low in zinc and cobalt but differed in their magnesium content. The metal composition of the cells was similar in both media, although several transition metal cations including Zn(II) and Co(II) were imported into *C. metallidurans* cells with a sevenfold higher initial transport rate in cells cultivated in the presence of 0.1 mM Mg(II) compared to 1 mM Mg(II) (57). As the metal composition was unchanged, the metal efflux rate could also be increased to compensate for the higher import rate (58). Alternatively, the import rate was down-regulated after some time, e.g., by flux control of the transporter activities (59). Nevertheless, this resulted in a different physiological state of cells grown in these two Co/Zn-starvation media despite the identical metal composition of the cells. Even more complicated, the need for FolE_IB2 in M1a and for FolE_IA in low metal medium could only be measured in the single mutants and was absent in the double mutants (Fig. 2 and 3). Moreover, all ∆*folE_IB2* mutants grew better in all media in the ∆*zupT* mutant; the ∆*zupT ∆folE_IA* and ∆*zupT ∆folE_IA* strains were not different in low zinc medium from the ∆*zupT* parent, but both grew with similar lag-phase delays compared to the parent in low metal medium (Fig. 4). Finally, FolE_IA was required for full growth in low zinc and low metal media in the ∆*zur* mutant (Fig. 5). This indicated some negative interferences between the activation of some FolEs under Co/Zn starvation conditions, which depend on the physiological state [cultivated in 1 or 0.1 mM Mg(II)], the Zn to Co ratio inside the cell, and components of the Zur regulon.

The Zur-repressed operon Op0317f_1 has a *cobW1* gene upstream of *folE_IB2* that encodes a member of the COG05203 protein family of GTP-dependent metal chaperones (Fig. S1). CobW1 binds to FolE_IB2 but only in the presence of zinc (4). As FolE_IB2 does not depend on zinc, this binding may prevent activation of FolE_IB2 in the presence of zinc because, here, FolE_IA provided sufficient FolE activity. Assuming that CobW1 also sequesters FolE_IB1 in a similar way, this would explain why the AE104 ∆*folE_IA* single mutant grew with a delayed lag-phase phenotype in low metal (M2) medium, but neither the double mutants nor the ∆*zur* ∆*folE_IA* mutant had this problem in low zinc or low metal media (M1a, M2). A preferential earlier release of FolE_IB2 by CobW1 than of FolE_IB1 would also explain the need for FolE_IB2 in the AE104 background in low zinc medium (M1a) and the growth advantages of the ∆*zupT ∆folE_IB2* mutants in all media. This hypothesis may indicate the importance of protein-protein interactions for metal homeostasis in *C. metallidurans*.

In summary, the FolEs are clearly the Achilles heel of metal homeostasis in *C. metallidurans*—and also act as sensors for the status of metal homeostasis. The bacterium possesses one FolE for every condition. FolE_IA is functional in environments with a high zinc and also high cadmium content, while FolE_IB1 is necessary when zinc becomes limiting. Iron or cobalt can also be used as cofactors with FolE_IB2, which solves the metal allocation issues under zinc and cobalt starvation conditions. The three FolEs also represent the final receptors of the metal allocation pathways. One pathway for zinc starts at the importer ZupT and delivers the metal to the RpoC subunit of the RNA polymerase and FolE_IA. A second pathway may deliver cobalt to the FolE_IBs, perhaps to reserve iron for other key enzymes. These pathways may overlap, and the components of the Zur regulon may be part of this or these pathways. Thus, by employing these FolE enzymes, *C. metallidurans* ensures THF synthesis is maintained in metal-replete as well as in metal-limiting environments.

## MATERIALS AND METHODS

### Bacterial strains and growth conditions

Plasmids and *C. metallidurans* strains (Table S13) in this study were all derivatives of the plasmid-free strain AE104 that lacks pMOL28 and pMOL30 (10). Tris-buffered mineral salts medium (10) containing 2 g sodium gluconate/L (TMM, named M1 in this publication) was used to cultivate these strains aerobically with shaking at 30°C. Medium M1a was M1 without trace element solution SL6 (60), M1b was M1 but only 0.1 mM MgCl_2_ instead of 1 mM, M2 was M1 without SL6 and with 0.1 mM MgCl_2_, and M3 was M1a without added iron ammonium citrate. Analytical grade salts of ZnCl_2_, CoCl_2_, and CdCl_2_ were used to prepare 1 M stock solutions, which were sterilized by filtration. Solid Tris-buffered media contained 20 g agar/L. SL6 (60) adds the following metals to the TMM: 35.3 nM Zn(II), 15.2 nM Mn(II), 485 nM borate, 84.1 nM Co(II), 5.87 nM Cu(II), 8.42 nM Ni(II), and 12.4 nM molybdate.

However, the actual metal content of the final TMM media was higher due to input of metals from the salts of the major bioelements, mainly the sodium sulfate source. As determined by inductively coupled plasma mass spectrometry, the metal contents of media M1 to M2 were 208 ± 15 µM calcium and 3.9 ± 0.2 µM iron. Routinely used M1 medium contained 953 ± 86 µM magnesium (1 mM added) and low magnesium media 85.2 ± 1.4 µM magnesium (0.1 mM added). Medium zinc media M1 and M1b contained 72.2 ± 13.7 nM zinc with 35.3 nM coming from the trace element solution SL6, 86.3 ± 17.3 nM cobalt (84.1 nM added), 157 ± 19 nM nickel (8.42 nM added), 48.9 ± 4.5 nM manganese (15.2 nM added), and 16.3 ± 14.0 nM copper (5.87 nM added). When the trace element solution was omitted, these values dropped to 35.2 ± 30.4 nM zinc, 34.9 ± 0.9 nM manganese, 89.1 ± 2.5 nM nickel, 14.3 ± 5.0 nM copper, and 4.6 ± 1.8 nM cobalt. While other salts provided a sufficient source for nickel, manganese, and copper, trace element solution SL6 was a major source for cobalt. Moreover, without SL6, the zinc content of the growth media varied strongly.

Growth curves in 96-well plates were conducted in medium zinc TMM (M1) if not mentioned otherwise. Always, a first pre-culture in medium zinc TMM was incubated at 30°C, 200 rpm up to early stationary phase, then diluted 1:20 into fresh medium, and incubated for 24 h at 30°C and 200 rpm to reach a late stationary phase. This second pre-culture was cultivated in the respective medium used for the main culture and used to inoculate parallel cultures, for dose-response curves with increasing metal concentrations, in 96-well plates (Greiner). Cells were cultivated for 24 h at 30°C and 1,300 rpm in a neoLab Shaker DTS-2 (neoLab, Heidelberg, Germany), and the optical density (OD) was determined at 600 nm as indicated in a TECAN infinite 200 PRO reader (Tecan Group Ltd., Männedorf, Switzerland).

### β-Galactosidase assay and *lacZ-*reporter constructions

The promoter-less *lacZ* reporter gene was inserted downstream of several target genes to construct reporter operon fusions. Alternatively, the target gene was disrupted by the *lacZ* insertion. In both cases, this was done by single cross-over recombination in *C. metallidurans* strains. A 300–400 bp PCR product of the 3′ end region of the respective target gene was amplified from total DNA of strain CH34, and the resulting fragments cloned into plasmid pECD794 (pLO2-*lacZ*) (61, 62). The respective operon fusion cassettes were inserted into the open reading frame of the target gene by conjugation and single cross-over recombination. *C. metallidurans* cells with a *lacZ* reporter gene fusion were cultivated as a pre-culture in TMM containing 1 g L^−1^ kanamycin at 30°C, 200 rpm until OD at 600 nm of 2 was reached, indicating the early stationary phase, diluted 20-fold into fresh medium, incubated with shaking at 30°C for 24 h to reach a late stationary phase, diluted 50-fold into fresh medium, and incubated with shaking at 30°C until a cell density of 100 Klett units was reached. This culture was distributed into sterile 96-well plates (Greiner Bio-One, Frickenhausen, Germany). After the addition of metal salts, incubation in the 96-well plates was continued for 3 h at 30°C in a neoLab Shaker DTS-2 (neoLab Migge Laborbedarf, Heidelberg, Germany). The turbidity at 600 nm was determined in a TECAN Infinite 200 Pro reader (TECAN, Männedorf, Switzerland), and the cells sedimented by centrifugation at 4°C for 30 min at 4,500 × *g*. The supernatant was discarded, and the cell pellets frozen at −20°C. For the enzyme assay, the pellet was suspended in 190 µL Z buffer (60 mM Na_2_HPO_4_, 40 mM NaH_2_PO_4_, 10 mM KCl, 1 mM MgSO_4_, 0.5 M beta-mercaptoethanol), and 10 µL permeabilization buffer was added (6.9 mM CTAB, cetyl-trimethyl-ammonium bromide, 12 mM sodium deoxycholate). The suspension was incubated with shaking at 30°C, and 20 µL ONPG solution (13.3 mM ortho-nitrophenyl-beta-D-galactopyranoside in Z-buffer without beta-mercaptoethanol) was added. Incubation was continued with shaking in a neoLab Shaker DTS-2 at 30°C until the yellow color of *o*-nitrophenol was clearly visible and stopped by the addition of 50 µL 1 M Na_2_CO_3_. The extinction at 420 and 550 nm was measured in a TECAN Infinite 200 Pro reader. The activity was determined as published (63) with a factor of 315.8 µM calculated from the path length of the 96-well plate and the extinction coefficient of *o*-nitrophenol:


$$activity=315.8\;µM*\{E_{420}-(1.75*E_{550})\}\;/\;reaction\;time$$

specific activity: activity divided by the cellular dry mass as published (63).

### Genetic techniques

Standard molecular genetic techniques were used (64, 65). For conjugative gene transfer, overnight cultures of donor strain *E. coli* S17/1 (66) and of the *C. metallidurans*-recipient strains grown at 30°C in Tris-buffered medium were mixed (1:1) and plated onto nutrient broth agar. After 2 d, the bacteria were suspended in TMM, diluted, and plated onto selective media as previously described (64).

Primer sequences are provided in Table S14. Plasmid pECD1003, a derivate of plasmid pCM184 (67), was used to construct deletion mutants. These plasmids harbor a kanamycin resistance cassette flanked by *loxP* recognition sites. Plasmid pECD1003 additionally carries alterations of 5 bp at each *loxP* site. Using these mutant *lox* sequences, multiple gene deletions within the same genome are possible without interferences by secondary recombination events (68, 69). Fragments of 300 bp upstream and downstream of the target gene were amplified by PCR, cloned into vector pGEM T-Easy (Promega), sequenced, and further cloned into plasmid pECD1003. The resulting plasmids were used in a double-cross-over recombination in *C. metallidurans* strains to replace the respective target gene by the kanamycin resistance cassette, which was subsequently also deleted by transient introduction of *cre* expression plasmid pCM157 (67). Cre recombinase is a site-specific recombinase from the phage P1 that catalyzes the *in vivo* excision of the kanamycin resistance cassette at the *loxP* recognition sites. The correct deletions of the respective transporter genes were verified by PCR. For construction of multiple deletion strains, these steps were repeated. The resulting mutants carried a small open reading frame instead of the wild-type gene to prevent polar effects.

### Purification of *Strep*-tagged FolE proteins

For production of all three FolE proteins, the *E. coli* strain BL21-pLysS was used. This strain contained plasmids pECD1672, pECD1611, or pECD1673, which were pASK-IBA3::*folE_IB1_*, pASK-IBA3::*folE_IB2_*, and pASK-IBA7::*folE_IA_*, respectively, and was cultivated in LB medium at 37°C with shaking until an optical density of between 0.5 and 1.0 at 600 nm was reached. Expression of the respective gene was induced by addition of 200 µg/L anhydrotetracycline, and incubation was continued for 3–4 h at 37°C. Cells were harvested by centrifugation (7,000 rpm, 20 min, SLA-3000 rotor, Sorvall) and suspended in buffer W. For the FolE_IB1 and FolE___IB2 proteins, 1 mM DTT was added. The cells were disrupted by sonication after addition of the protease inhibitor PMSF (1 mM) and DNaseI (10 µg/mL). The soluble fraction was obtained by centrifugation (30 min, 14,000 rpm, Eppendorf 5417R or 20 min, 17,000 rpm, SS-34 rotor, Sorvall), and the proteins were purified using the Strep-Tactin affinity column by following the manufacturer’s protocol (IBA GmbH) but without EDTA. If it was necessary, the protein was concentrated by using Vivaspin (6 or 20) columns (molecular weight cut of 10,000 or 30,000; polyethersulfone membrane; Sartorius Stedim Biotech, Göttingen, Germany). The concentration was determined by using a NanoPhotometer (IMPLEN) or Tecan Spark Multiplate Reader with a NanoQuant-plate. The required extinction coefficient *ε*_280_ and the size of the N-or C-terminal *Strep-*tagged FolE-proteins were calculated by using the ProtParam tool of the ExPASy server (https://web.expasy.org/protparam). The respective extinction coefficients *ε*_280_ in M^−1^ cm^−1^ were 26,720 (N-*Strep*-FolE_IA, 31513.91 Da), 21,555 (FolE_IB1-C-*Strep*, 32259.71 Da), and 39,210 (FolE_IB2-C-*Strep*, 38153.56 Da). Protein quality was analyzed by polyacrylamide gel electrophoresis in the presence of sodium dodecyl sulfate (12.5%, wt/vol) followed by Coomassie brilliant blue and Western blotting using a Strep-Tactin HRP-conjugate (Bio-Rad, USA).

### GTP-cyclohydrolase I activity assay

The assay was performed according to Sankaran et al. (28), but Tris-buffer was used instead of HEPES. The reaction buffer (100 mM Tris, pH = 7) contained 100 mM KCl, the appropriate amount of other metal salts (MnCl_2,_ CoCl_2_, MgCl_2_, FeSO_4_, CdCl_2_, NiCl_2_, and ZnCl_2_), and 1 mM DTT. After 4 µM of the respective FolE protein had been added, the reaction was started with GTP at a final concentration of 1 mM and a final volume of 100 µL. The conversion of GTP to 7,8 -dihydroneopterin triphosphate was monitored every 2 min for 1–2 h at 30°C at 330 nm, the absorption maximum of the product, in a Tecan Spark multiplate reader. Negative controls did not contain protein. The specific activity (µmol min^−1^ g^−1^_protein_) was calculated from the slope of the time-dependent increase in the absorption at 330 nm between 10 and 30 min, the extinction coefficient of 7,8 -dihydroneopterin triphosphate, the diameter of the reaction cell, and the protein amount in the sample. Reconstitution of the FolE_IBs with 1 mM Fe(II)SO_4_ was performed in an anaerobic hood. The samples were kept anaerobically and measured under a constant flow of molecular nitrogen.

### Inductively coupled plasma mass spectrometry

Cells were incubated in TMM for 20 h at 30°C shaking at 200 rpm, diluted 20-fold into fresh TMM medium, and continued shaking at 30°C for 24 h. Cells were diluted 50-fold into fresh medium containing added copper or not, and continued shaking at 30°C at 200 rpm until 150 Klett was reached (mid-exponential phase of growth). Ten milliliters of the cells were harvested by centrifugation, washed twice with 50 mM TrisHCl buffer (pH 7.0) containing 50 mM EDTA at 0°C, and suspended in 50 mM TrisHCl buffer (pH 7.0). For ICP-MS analysis, HNO_3_ (trace metal grade; Normatom/PROLABO) was added to the samples to a final concentration of 67% (wt/vol), and the mixture mineralized at 70°C for 2 h. Samples were diluted to a final concentration of 2% (wt/vol) nitric acid. Indium and germanium were added as internal standards at a final concentration of 1 and 10 ppb each. Elemental analysis was performed via ICP-MS using Cetac ASX-560 sampler (Teledyne, Cetac Technologies, Omaha, Nebraska), a MicroFlow PFA-100 nebulizer (Elemental Scientific, Mainz, Germany), and an ICAP-RQ ICP-MS instrument (Thermo Fisher Scientific, Bremen) operating with a collision cell and flow rates of 4.5 mL × min^−1^ of He/H_2_ [93%/7% (70)], with an Ar carrier flow rate of 0.76 L × min^−1^ and an Ar make-up flow rate at 15 L × min^−1^. An external calibration curve was recorded with ICP-multi-element standard solution XVI (Merck) in 2% (vol/vol) nitric acid. The sample was introduced via a peristaltic pump and analyzed for its metal content. For blank measurement and quality/quantity thresholds, calculations based on DIN32645 TMM were used. The results were calculated from the ppb data as atoms per cell as described (14).

### Inductively coupled plasma mass spectrometry of the purified proteins

For ICP-MS analysis, HNO_3_ (trace metal grade; Normatom/PROLABO) was added to the samples to a final concentration of 67% (wt/vol), and the mixture mineralized at 70°C for 2 h. Samples were diluted to a final concentration of 5% (wt/vol) nitric acid and up to 1 mg/mL of the respective protein. Indium and germanium were added as internal standards at a final concentration of 1 and 10 ppb each. Elemental analysis was performed via ICP-MS using Cetac ASX-560 sampler (Teledyne, Cetac Technologies, Omaha, Nebraska), a MicroFlow PFA-200 nebulizer (Elemental Scientific, Mainz, Germany), and an ICAP-TQ ICP-MS instrument (Thermo Fisher Scientific, Bremen) operating with a collision cell and flow rates of 4.5 mL × min^−1^ of He/H_2_ [93%/7% (70)], with an Ar carrier flow rate of 0.76 L × min^−1^ and an Ar make-up flow rate at 15 L × min^−1^. An external calibration curve was recorded with ICP-multi-element standard solution XVI (Merck) in 2% (vol/vol) nitric acid. The sample was introduced via a peristaltic pump and analyzed for its metal content. For blank measurement and quality/quantity thresholds, calculations based on DIN32645 TMM were used. The results were calculated from the ppb data as mole metal per mole protein or per cell as described (14).

### Statistics

Students’ *t*-test was used, but in most cases, the distance value, *D*, has been used several times previously for such analyses (12, 71, 72). It is a simple, more useful value than Student’s *t*-test because non-intersecting deviation bars of two values (*D* >1) for three repeats always means a statistically relevant (≥ 95%) difference, provided the deviations are within a similar range. At *n* = 4, significance is ≥97.5%; at *n* = 5, ≥99% (significant); and at *n* = 8, ≥99.9% (highly significant).

## SUPPLEMENTAL MATERIAL

Supplemental material jb.00395-23-s0001.pdf Tables S1 to S14; Figures S1 to S12.

ASM does not own the copyrights to Supplemental Material that may be linked to, or accessed through, an article. The authors have granted ASM a non-exclusive, world-wide license to publish the Supplemental Material files. Please contact the corresponding author directly for reuse.
