# Supplementary material for: Protecting the Achilles heel: three FolE_I-type GTP-cyclohydrolases needed for full growth of metal-resistant Cupriavidus metallidurans under a variety of conditions
Source: J Bacteriol. 2024 Jan 16;206(2):e00395-23. doi: 10.1128/jb.00395-23 (PMC10882993; doi:10.1128/jb.00395-23)
Supplement: Supplemental material — Tables S1 to S14; Figures S1 to S12. [file jb.00395-23-s0001.pdf]

Supplementary Data

Supplementary Table S1. Activity of beta-galactosidase fusions of *glyA* in AE104 strain backgrounds Δ*zupT* and Δ*zur*, and in the presence or absence of 50 µM Zn (II), inhibitors of the THF biosynthesis or 5 g/L yeast extract[a].

strain	addition				
	none	YE, 5 g/L	TMP, 0.5 mg/L	SUAM, 0.2 g/L	Zn(II), 50 µM
AE104	177 ± 18.5	146 ± 30.8	**236 ± 29.5**	167 ± 6.20	165 ± 17.4
AE104 Δ*folE_IA*	167 ± 18.8	138 ± 42.1	236 ± 29.2	165 ± 5.83	167 ± 8.58
AE104 Δ*folE_IB1*	208 ± 17.9	**229 ± 26.6**	280 ± 28.1	214 ± 7.25[b]	175 ± 29.0[c]
Δ*zupT*	156 ± 27.7	147 ± 31.4	224 ± 21.2	169 ± 22.5	155 ± 20.6
Δ*zupT* Δ*folE_IA*	176 ± 25.7	184 ± 23.4	239 ± 20.5	177 ± 19.6	128 ± 22.8
Δ*zupT* Δ*folE_IB1*	207 ± 28.6	**260 ± 24.7**	239 ± 7.33	241 ± 3.11[b]	191 ± 25.1
Δ*zur*	**128 ± 15.5**	n.d.	**156 ± 34.3**	n.d.	n.d.
Δ*zur* Δ*folE_IA*	132 ± 24.1	n.d.	166 ± 35.9	n.d.	n.d.
Δ*zur* Δ*folE_IB1*	123 ± 25.1	n.d.	157 ± 17.9	n.d.	n.d.
Δ*zur* Δ*folE_IB2*	123 ± 19.7	n.d.	151 ± 33.5	n.d.	n.d.

[a]The strains were cultivated in medium zinc TMM (M1) with or without additives at the indicated concentrations (YE, yeast extract). Bold faced number: different (D > 1) in the comparisons (i) AE104 with/without additions; (ii) Δ*zupT*, Δ*zur* compared to AE104; (iii) other mutants compared to their respective parent under the same condition. With the exception of three experiments, n ≥ 3 biological repeats, mean values and deviations indicated; n.d., not done.
[b]The result of one biological replicate only, mean values and deviations from the technical repeats. Not further pursued because of the lack of any difference
[c]The results of two biological replicates, mean values and deviations from the technical and biological repeats. Also not pursued because of the lack of any difference compared to the control.

Supplementary Table S2. Activity *folE_IA* and *folE_IB1* fusions in the presence or absence inhibitors of the THF biosynthesis[a].

		Fusion	
Strain	Addition	*folE_IA::lacZ*	*folE_IB1::lacZ*
AE104	none	91.1 ± 16.4	117 ± 17.3
	SUAM, 0.2 g/L	83.9 ± 15.1	121 ± 15.1
	SUAM, 0.75 g/L	97.3 ± 25.0	105 ± 27.7
	TMP, 0.5 mg/L	89.8 ± 15.5	122 ± 10.3
	TMP, 2 mg/L	86.1 ± 11.8	101 ± 18.8
Δ*zupT*	none	71.6 ± 16.0	116 ± 23.5
	SUAM, 0.2 g/L	88.3 ± 20.2	122 ± 24.0
	SUAM, 0.75 g/L	61.3 ± 14.4	n.d.
	TMP, 0.5 mg/L	89.9 ± 15.0	137 ± 19.8
	TMP, 2 mg/L	76.9 ± 13.5	101 ± 24.0
Δ*zur*	none	92.3 ± 10.6	113 ± 19.2
	SUAM, 0.75 g/L	88.8 ± 1.70	96.3 ± 27.2
	TMP, 2 mg/L	81.6 ± 16.4	107 ± 18.9

[a]The strains were cultivated in medium zinc TMM (M1) with or without sulfonamide (SUAM) or trimethoprim (TMP) at the indicated concentrations. Both inhibit later steps of the tetrahydrofolate (THF) biosynthesis, the dihydropteroate synthase and dihydrofolate reductase, respectively; n ≥ 3, deviations shown

Supplementary Table S3. Activity of *folE_IA* and *folE_IB1* fusions in the presence or absence of metal chelators[a].

Strain	Addition			
	none	EDTA	DIP	TPEN
<u>Ω(*folE_IA*+::*lacZ*+)</u>				
AE104	91.1 ± 16.4	101 ± 13.6	107 ± 46.0	102 ± 9.59
Δ*zupT*	71.6 ±16	88.3 ± 19.1	73.2 ± 15.5	n.d.
Δ*zur*	92.3 ±10.6	92.2 ± 10.2	84.5 ± 13.6	93.4 ± 7.14
AE104 Δ*folE_1B1*	**126 ± 8.13**	124 ± 13.3	147 ± 26.6	125 ± 17.6
<u>Ω(*folE_IB1*+::*lacZ*+)</u>				
AE104	117 ± 17.3	96.6 ± 21.8	**81.1 ± 14.7**	116 ± 18.4
Δ*zupT*	116 ± 23.5	119 ± 19.6	n.d.	n.d.
Δ*zur*	113 ±19.2	120 ± 21.8	112 ± 16.0	112 ± 10.2
AE104 Δ*folE_1A*	**200 ± 20.8**	**193 ± 21.5**	**201 ± 29.5**	**182 ± 25.5**

[a]The strains were cultivated in medium zinc TMM (M1) with or without 100 µM ethylene diamine tetraacetate (EDTA), 10 µM dipyridyl (DIP, also bipyridyl) or 1 µM *N*,*N*,*N′*,*N′*-tetrakis(2-pyridinylmethyl)-1,2-ethanediamine (TPEN) and the specific beta-galactosidase activity was determined. Bold-faced, if different (D>1) in the comparisons (i) AE104 parent with additions to no additions or (ii) mutants to parent AE104 under the same conditions; n.d. not done; n ≥ 3, deviations shown.

Supplementary Table S4. Metal content of *C. metallidurans* strain AE104 and mutants with deletions in the genes for FolE_I-type enzymes after cultivation under iron and zinc starvation conditions [a].

Bacterial strain		Mg x 10^6	Fe x 10^4	Zn x 10^3	Co x 10^2	Ni x 10^2	Mn x 10^2
AE104	M1 mZn	12.7±2.3	85.9±19.5	68.8±17.3	268±72	23.6±8.46	3.94±2.13
	M3 lFe	**7.53±0.99**	**18.3±6.47**	**23.8±3.57**	**11.4±3.0**	15.4±5.06	**27.0±6.5**
Δ*folE_IA*	M1 mZn	12.6±1.6	85.5±13.3	71.5±18.5	204±74	15.5±5.28	6.98±5.03
	M3 lFe	9.59±0.37	**14.2±1.63**	**35.0±7.17**	**13.2±4.8**	13.1±1.96	**33.5±4.2**
Δ*folE_IB1*	M1 mZn	14.1±4.3	81.7±16.9	78.0±13.7	243±85	19.7±5.68	6.40±3.46
	M3 lFe	**6.55±0.42**	**11.8±1.23**	**24.4±3.01**	**10.7±3.3**	12.0±5.12	**20.3±2.7**
Δ*folE_IB2*	M1 mZn	13.3±3.0	83.6±18.3	84.3±14.6	262±70	19.4±3.44	4.61±2.00
	M3 lFe	**6.56±0.42**	**13.4±2.33**	**25.7±2.71**	**11.9±3.4**	**8.98±1.43**	**21.7±2.6**
Δ*zupT*	M1 mZn	12.3±1.59	91.7±14.4	**33.1±12.4**	113±28.0	36.7±3.90	1.25±0.30
	M3 lFe	12.5±0.13	**44.9±0.36**	53.7±1.22	**22.2±0.0**	44.0±1.36	**59.4±0.9**
Δ*zupT* Δ*folE_IA*	M1 mZn	14.1±2.05	87.4±13.7	**36.0±10.3**	113±35.3	26.7±12.2	2.93±1.81
	M3 lFe	10.1±1.39	**25.2±5.46**	**27.3±5.96**	**11.8±2.5**	**11.7±3.30**	**27.6±9.3**
Δ*zupT* Δ*folE_IB1*	M1 mZn	13.9±1.05	93.3±12.4	**45.9±3.75**	88.2±29.4	21.5±13.1	2.98±1.95
	M3 lFe	12.0±1.56	**33.2±8.09**	55.8±23.1	**17.2±3.8**	17.3±5.08	**30.7±2.7**
Δ*zupT* Δ*folE_IB2*	M1 mZn	12.8±1.39	94.5±19.1	**34.1±7.31**	75.4±13.4	17.3±7.99	1.69±0.54
	M3 lFe	11.3±2.90	**29.3±6.37**	42.9±10.8	**12.8±2.4**	11.7±3.30	**27.6±9.3**

[a]The metal content in metals per cell was determined with the ICP-MS of *C. metallidurans* strain AE104 and its Δ*folE_IA,* Δ*folE_IB1* and Δ*folE_IB2* mutant in medium zinc TMM (M1, mZn) or low iron TMM (M3, lFe, no SL6 and no iron)). The values for M1 are the same as in Table 5 and listed here again to facilitate the comparison. Bold, difference to the AE104 value in M1 with D>1. Three biological repeats, deviations indicated, n.a., not analyzed.

Supplementary Table S5. Growth rates of *C. metallidurans* strains under various conditions (1/h)[a]

Strain	Growth medium			
	M1, mZn	M1a, lZn	M1b, lMg	M2, lM
AE104	0.30 ± 0.01	0.29 ± 0.02	**0.33 ± 0.01**	***0.27 ± 0.01***
AE104 Δ*folE_IA*	0.30 ± 0.02	0.30 ± 0.01	0.30 ± 0.02	0.24 ± 0.02
AE104 Δ*folE_IB1*	0.31 ± 0.01	0.31 ± 0.01	0.36 ± 0.02	0.28 ± 0.01
AE104 Δ*folE_IB2*	0.29 ± 0.01	0.30 ± 0.02	0.33 ± 0.02	0.26 ± 0.02
AE104 Δ*folE_IB2* Δ*folE_IB1*	0.30 ± 0.03	0.31 ± 0.03	0.31 ± 0.01	0.27 ± 0.01
AE104 Δ*folE_IB2* Δ*folE_IA*	0.29 ± 0.02	0.25 ± 0.04	0.31 ± 0.01	0.29 ± 0.02
AE104 Δ*folE_1A* Δ*folE_1B1::dis*	0.28 ± 0.03	0.31 ± 0.02	0.16 ± 0.07	0.20 ± 0.02
Δ*zupT*	0.27 ± 0.02	0.27 ± 0.02	0.34 ± 0.03	0.25 ± 0.04
Δ*zupT* Δ*folE_IA*	0.26 ± 0.01	0.29 ± 0.01	0.30 ± 0.01	0.22 ± 0.03
Δ*zupT* Δ*folE_IB1*	0.23 ± 0.04	0.23 ± 0.02	0.25 ± 0.02	0.19 ± 0.04
Δ*zupT* Δ*folE_IB2*	0.32 ± 0.05	0.33 ± 0.03	0.32 ± 0.03	0.34 ± 0.04
Δ*zur*	**0.35 ± 0.02**	**0.35 ± 0.02**	**0.38 ± 0.04**	**0.33 ± 0.03**
Δ*zur* Δ*folE_IA*	0.32 ± 0.02	0.33 ± 0.01	***0.31 ± 0.01***	0.29 ± 0.01
Δ*zur* Δ*folE_IB1*	0.34 ± 0.01	0.36 ± 0.02	0.34 ± 0.04	0.33 ± 0.03
Δ*zur* Δ*folE_IB2*	0.35 ± 0.01	0.36 ± 0.02	0.36 ± 0.03	0.33 ± 0.03

[a]The growth rates were calculated from the data shown in Figs. 3 to 6. Increased growth rates bold-faced, decreased growth rates in bold-faced and italic letters, both if D>1. Compared were (i) AE104 parent in low zinc (M1a, lZn), low magnesium (M1b, lMg) and low zinc and magnesium (M2, lM) to AE104 parent in medium zinc (M1, mZn); (ii) the mutants Δ*zupT* and Δ*zur* to parent AE104 in the same medium, and (iii) the *folE* mutant to the respective parent strain (AE104, Δ*zupT* or Δ*zur*) in the same medium.

Supplementary Table S6. Metal content of *C. metallidurans* strain AE104 and mutants with deletions in the genes for FolE_I-type enzymes after cultivation under metal starvation conditions [a].

Strain	Mg $\times 10^6$	Fe $\times 10^4$	Zn $\times 10^3$	Co $\times 10^2$	Ni $\times 10^2$	Mn $\times 10^2$
	<u>No addition</u>					
AE104	12.7±2.3	85.9±19.5	68.8±17.3	268±7.2	23.6±8.46	3.94±2.13
Δ*zupT*	12.3±1.59	91.7±14.4	**33.1±12.4**	**113±28.0**	36.7±3.90	1.25±0.30
	<u>0.1 mM EDTA</u>					
AE104	10.6±0.68	71.3±14.2	54.4±15.3	**83.1±34.4**	n.d.	3.88±3.02
AE104 Δ*folE_IA* [b]	12.9±0.10	78.8±0.08	53.1±0.66	**146±0.27**	n.d.	1.56±0.58
AE104 Δ*folE_IB1* [b]	13.1±0.26	69.6±1.57	47.1±1.32	**158±2.43**	n.d.	1.07±0.91
AE104 Δ*folE_IB2* [b]	12.9±0.11	64.0±0.07	47.2±0.78	**115±0.78**	n.d.	2.39±0.34
Δ*zupT*	13.5±3.37	88.9±30.0	**22.9±7.88**	**86.6±20.7**	34.0±10.5	n.d.
Δ*zupT* Δ*folE_IA*	14.2±1.89	68.9±11.1	**19.9±4.47**	**78.6±5.88**	14.0±5.06	4.39±0.68 [c]
Δ*zupT* Δ*folE_IB1*	13.8±0.67	68.8±6.14	**22.7±1.43**	**70.6±7.57**	13.4±7.65	1.12±0.73 [d]
Δ*zupT* Δ*folE_IB2*	12.7±1.41	80.0±12.9	**17.6±7.16**	**49.4±12.3**	7.47±1.35 [d]	4.36±4.48 [d]
	<u>25 µM DIP</u>					
AE104	15.1±0.96	84.5±2.68	77.0±3.39	**33.7±10.5**	n.d.	n.d.
AE104 Δ*folE_IA*	14.4±0.91	89.6±10.9	84.2±8.47	**46.7±24.8**	n.d.	n.d.
AE104 Δ*folE_IB1*	12.9±2.25	89.5±4.00	70.9±15.6	**83.1±33.5**	n.d.	n.d.
AE104 Δ*folE_IB2*	17.0±1.28	87.5±10.6	79.0±4.75	**77.6±25.8**	n.d.	n.d.

[a]The metal content in metals per cell was determined with the ICP-MS of *C. metallidurans* strain AE104, Δ*zupT* and its Δ*folE_IA,* Δ*folE_IB1* and Δ*folE_IB2* mutant in medium zinc TMM (M1) in the presence of either 0.1 mM EDTA or 25 µM DIP. The metal content without additions is given for reference. Bold-faced numbers are different (D > 1) from the AE104 value; n.d., no data since below the detection limit. Three biological repeats, deviations indicated. Exceptions: [b]no differences and therefore not repeated; [c]one result, repeats below detection limit, [d] two results, one repeat below detection limit, therefore not bold-faced despite the difference to the AE104 value.

Supplementary Table S7. Growth rates (h^{-1}) in the presence of 100 µM EDTA

Strain	No addition	EDTA, 100 µM
AE104 (WT)	0.30 ± 0.01	0.30 ± 0.02
AE104 Δ*folE_IA*	0.30 ± 0.02	0.30 ± 0.02
AE104 Δ*folE_IB1*	0.31 ± 0.01	0.34 ± 0.03
AE104 Δ*folE_IB2*	0.29 ± 0.01	0.31 ± 0.04
Δ*zupT*	0.27 ± 0.02	0.22 ± 0.05
Δ*zupT* Δ*folE_IA*	0.26 ± 0.01	0.28 ± 0.02
Δ*zupT* Δ*folE_IB1*	0.23 ± 0.04	0.24 ± 0.04
Δ*zupT* Δ*folE_IB2*	0.32 ± 0.05	0.24 ± 0.01

Supplementary Table S8. IC_{50} values for TPEN and DIP

Strain	TPEN, (µM)	DIP, (µM)
AE104	1.1 ± 0.4	57 ± 4
AE104 Δ*folE_IA*	1.2 ± 0.2	**36 ± 7**
AE104 Δ*folE_IB1*	1.3 ± 0.3	63 ± 8
AE104 Δ*folE_IB2*	0.9 ± 0.3	62 ± 3

Bold faced number: different (D > 1) from the respective AE104 value.

Supplementary Table S9. IC_{50} values for SUAM and TMP

Strain	SUAM, (g/L)	TMP, (mg/L)
AE104	2.0 ± 0.5	4.0 ± 0.2
AE104 Δ*folE_IA*	2.0 ± 0.5	3.7 ± 0.3
AE104 Δ*folE_IB1*	2.1 ± 0.4	**3.3 ± 0.2**
AE104 Δ*folE_IB2*	1.9 ± 0.6	4.2 ± 0.2
AE104 Δ*folE_IB2* Δ*folE_IA*	**0.9 ± 0.1**	4.2 ± 0.2
AE104 Δ*folE_IB* Δ*folE_IB1*	**1.0 ± 0.2**	**2.8 ± 0.2**
Δ*zupT*	**0.9 ± 0.2**	3.6 ± 0.3
Δ*zupT* Δ*folE_IA*	1.2 ± 0.3	3.1 ± 0.6
Δ*zupT* Δ*folE_IB1*	**0.8 ± 0.2**	**2.0 ± 0.4**
Δ*zupT* Δ*folE_IB2*	1.9 ± 0.5	3.7 ± 0.3

Bold faced number: different (D > 1) from the respective AE104 value.

Supplementary Table S10. IC_{50} values for cadmium, cobalt, paraquat (PQ) and hydrogen peroxide

Strain	Cd(II), (µM)	Co(II), (µM)	PQ, (µM)	H_2O_2, (mM)
AE104	82 ± 14	173 ± 25	181 ± 17	9.4 ± 0.9
AE104 Δ*folE_IA*	**12 ± 3**	**81 ± 6**	**101 ± 13**	**5.4 ± 1.5**
AE104 Δ*folE_IB1*	74 ± 11	173 ± 43	214 ± 11	9.7 ± 1.4
AE104 Δ*folE_IB2*	104 ± 8	196 ± 17	165 ± 18	9.4 ± 0.5
Δ*zupT*	**5.4 ± 2.0**	**32 ± 15**	149 ± 21	9.2 ± 1.5
Δ*zupT* Δ*folE_IA*	6.3 ± 1.4	38 ± 11	**62 ± 17**	**3.7 ± 2.0**
Δ*zupT* Δ*folE_IB1*	2.5 ± 0.9	65 ± 19	93 ± 31	7.9 ± 2.2
Δ*zupT* Δ*folE_IB2*	6.4 ± 2.8	**170 ± 25**	108 ± 9	9.5 ± 1.8
Δ*zur*	**126 ± 12**	169 ± 32	206 ± 8	8.1 ± 1.8
Δ*zur* Δ*folE_IA*	139 ± 11	108 ± 38	197 ± 16	10 ± 3
Δ*zur* Δ*folE_IB1*	135 ± 11	212 ± 20	205 ± 12	6.8 ± 0.6
Δ*zur* Δ*folE_IB2*	132 ± 11	207 ± 26	195 ± 9	7.9 ± 2.1

Bold faced number: different (D > 1) from the respective value of the parent AE104 or Δ*zupT*.

Supplementary Table S11. Metal content of *C. metallidurans* strain AE104 and mutants with deletions in the genes for FolE_I-type enzymes after cultivation in the presence of 1 µM Cd(II) [a].

Strain	Cd, (µM)	Mg x 10^6	Fe x 10^4	Zn x 10^3	Co x 10^2	Ni x 10^2	Mn x 10^2	Cd x 10^3
AE104	0	12.7±2.3	85.9±19.5	68.8±17.3	268±72	23.6±8.46	3.94±2.13	0.18±0.07
AE104	1	12.0±1.7	79.56±11.6	55.5±8.8	**57.9±24.5**	14.6±5.7 [c]	1.94±0.23[b]	**42.2±8.6**
AE104 Δ*folE_IA*	1	9.1±0.9	71.8±6.7	**38.3±3.8**	**79.4±13.2**	7.70±0.55 [c]	1.37±0.97[b]	**46.4±6.5**
AE104 Δ*folE_IB1*	1	9.1±0.9	66.7±8.2	43.0±8.3	**65.4±13.1**	10.7±2.9 [c]	1.23±0.36[b]	**41.0±5.1**
AE104 Δ*folE_IB2*	1	9.0±0.6	66.5±2.1	48.5±2.3	**39.1±.6.26**	10.6±2.39 [c]	0.61±0.24[b]	**30.8±2.6**
Δ*zupT*	0	12.3±1.59	91.7±14.4	**33.1±12.4**	**113±28.0**	36.7±3.9	1.25±0.30	1.3±0.05
Δ*zupT*	1	11.0±0.93	80.2±3.74	23.8±1.4	**70.6±15.2**	48.1±22.9	0.81±0.22	**72.8±0.7**
Δ*zupT* Δ*folE_IA*	1	18.1±2.96	111±16.0	49.6±1.1	**103±20.9**	26.2±3.2	7.40±1.38	**89.4±22**
Δ*zupT* Δ*folE_IB1*	1	16.0±4.81	110±23.2	36.0±10.1	**92.3±9.83**	15.6±4.1	3.94±3.54	**92.3±36**
Δ*zupT* Δ*folE_IB2*	1	11.5±1.42	83.9±11.6	26.0±3.4	**61.9±8.12**	26.1±25	1.29±0.37	**53.6±12**

[a]The metal content in metals per cell was determined with the ICP-MS of *C. metallidurans* strain AE104, Δ*zupT* and its Δ*folE_IA,* Δ*folE_IB1* and Δ*folE_IB2* mutant in medium zinc TMM (M1) in the presence of 1 µM Cd(II). The metal content without additions is given for reference. Bold-faced numbers are different (D > 1) from the value of the respective parent; n.d., no data since below the detection limit. Three biological repeats, deviations indicated. Exceptions: [b]one result, repeats below detection limit, [c] two results, one repeat below detection limit, therefore not bold-faced despite the difference to the AE104 value.

Supplementary Table S12. Presence of the purine and THF *de novo* biosynthesis pathway in *C. metallidurans*[a].

Locus tag[b]	Gene[c]	Operon[d]	Description[e]
Purine Biosynthesis			
Rmet_0289	***prs***	**Op0084r_3**	Q1LRQ1 Ribose-phosphate pyrophosphokinase
Rmet_2460	***purF***	**Op0688r_6**	Q1LKI9 Amidophosphoribosyltransferase
Rmet_0784	***purD***	Op0222r_1	Q1LQA6 Phosphoribosylamine--glycine ligase
**Rmet_2878**	***purN***	**Op0808r_1**	Q1LJC5 Phosphoribosylglycinamide formyltransferase
Rmet_1820	***purT***	**Op0527f_2**	**Q1LMC7 Phosphoribosylglycinamide formyltransferase 2; Q = 3.25**
Rmet_1870	***purL***	Op0540r_2	Q1LM77 Phosphoribosylformylglycinamidine synthase
Rmet_2911	***purM***	**Op0824r_1**	Q1LJ92 Phosphoribosylformylglycinamidine cyclo-ligase
Rmet_0506	***purK***	**Op0135f_1**	Q1LR34 Phosphoribosylaminoimidazole carboxylase
Rmet_0505	***purE***	**Op0135f_1**	Q1LR35 Phosphoribosylaminoimidazole carboxylase, catalytic subunit
Rmet_0504	***purC***	**Op0135f_1**	Q1LR36 Phosphoribosylaminoimidazole-succinocarboxamide synthase
Rmet_2956	***purB***	**Op0832r_1**	Q1LJ47 Adenylosuccinate lyase
**Rmet_0427**	***purH***	**Op0116r_3**	Q1LRB3 Bifunctional purine biosynthesis protein purH
Rmet_1461	***guaB***	**Op0409f_1**	Q1LND2 Inosine-5'-monophosphate dehydrogenase
Rmet_1463	***guaA***	**Op0409f_1**	Q1LND0 GMP synthase (Glutamine-hydrolyzing)
Rmet_0856	***gmk***	Op0245f_1	Q1LQ34 Guanylate kinase
Rmet_0532	***adk***	**Op0148r_1**	Q1LR08 Adenylate kinase
Rmet_2110	***ndk***	**Op0608r_1**	Q1LLI7 Nucleoside diphosphate kinase
THF Biosynthesis			
Rmet_3990	***folE_IA***	Op1119r_2	Q1LG68 GTP cyclohydrolase IA, chromid
Rmet_2614	***folE_IB1***	Op0732r_2	Q1LK35 GTP cyclohydrolase IB1
Rmet_1099	*folE_IB2*	*Op0317f_1*	*Q1LPE1 GTP cyclohydrolase IB2, Q = 1.84*
Rmet_0380	***ntpA***	Op0103f_5	Q1LRG0 NUDIX hydrolase
Rmet_0183	***folB***	Op0055f_1	Q1LS07 Dihydroneopterin aldolase
Rmet_2915	***folK***	Op0825f_2	Q1LJ88 2-amino-4-hydroxy-6-hydroxymethyldihydropteridine pyrophosphokinase
Rmet_2187	***folP***	**Op0628r_2**	Q1LLB0 Dihydropteroate synthase
Rmet_2463	***folC***	**Op0688r_4**	Q1LKI6 FolC bifunctional protein
Rmet_2569	***folA***	**Op0711f_1**	Q1LK80 Dihydrofolate reductase
C1-loading			
Rmet_4537	***serA2***	*Op1298f_1*	Q1LEM4 D-isomer specific 2-hydroxyacid dehydrogenase, NAD-binding, chromid
Rmet_0715	***serC***	**Op0203f_2**	Q1LQH5 Phosphoserine aminotransferase
Rmet_1368	***serB***	Op0384r_1	Q1LNM5 Phosphoserine phosphatase
**Rmet_2680**	***glyA***	**Op0757f_1**	Q1LJX3 Serine hydroxymethyltransferase
**Rmet_3480**	***gcvT***	**Op0969f_1**	Q1LHM4 Aminomethyltransferase
Rmet_3481	***gcvH***	**Op0969f_2**	Q1LHM3 Glycine cleavage system H protein
Rmet_3482	***gcvP***	**Op0969f_2**	***Q1LHM2 Glycine dehydrogenase (decarboxylating), Q = 0.40***
C1-interconversion			
**Rmet_1192**	***folD***	**Op0344r_1**	Q1LP48 Bifunctional protein folD
**Rmet_0175**	*fau*	**Op0052r_1**	Q1LS15 5-formyltetrahydrofolate cyclo-ligase
**Rmet_2988**	*metF2*	**Op0842r_2**	A0HB18 Methylenetetrahydrofolate reductase
**Rmet_0172**	***metF***	Op0051f_1	Q1LS18 Methylenetetrahydrofolate reductase

Important sinks			
**Rmet_0088**	***metH***	Op0025f_1	Q1LSA2 Methionine synthase (B12-dependent)
**Rmet_2568**	***thyA***	**Op0711f_1**	Q1LK81 Thymidylate synthase
**Rmet_3564**	***fmt***	Op0988r_1	Q1LHE0 Methionyl-tRNA formyltransferase
**Rmet_2917**	***panB***	Op0825f_3	Q1LJ86 3-methyl-2-oxobutanoate hydroxymethyltransferase

[a]The data were compiled from data base submissions connected to several publications (1-3) and EcoCyc (4). [b]**Locus tag bold: uses THF**; [c]**gene name bold**, gene products were identified, quantified, and more than 100 copies per cell were found (2). [d](3). [e]Q, Q(AE104: EDTA/Zn), regulation of the respective gene in EDTA-treated *C. metallidurans* AE104 cells compared to those cultivated in the presence of zinc (1), **bold up-** and **bold-*italics*** *down*-regulated. For the operons, under RpoD control bold, not under RpoD control on a grey field, both (two promoters) bold and on a grey field, weak RpoD motif normal, no transcriptional start site identified in italics (5).

Supplementary Table S13. Bacterial strains

Strain	Description	Reference
AE104	Plasmid-free derivative of *C. metallidurans* strain CH34	(6)
DN 955	AE104 Δ*Rmet_1099 (folE_1B2)*	This work
DN 956	AE104 Δ*Rmet_2614 (folE_1B1)*	This work
DN 957	AE104 Δ*Rmet_3990 (folE_1A)*	This work
DN 958	AE104 Δ*zupT* Δ*Rmet_1099 (folE_1B2)*	This work
DN 959	AE104 Δ*zupT* Δ*Rmet_2614 (folE_1B1)*	This work
DN 960	AE104 Δ*zupT* Δ*Rmet_3990 (folE_1A)*	This work
DN 961	AE104 Δ*zur* Δ*Rmet_1099 (folE_1B2)*	This work
DN 962	AE104 Δ*zur* Δ*Rmet_2614 (folE_1B1)*	This work
DN 963	AE104 Δ*zur* Δ*Rmet_3990 (folE_1A)*	This work
DN 968	AE104 Δ*Rmet_3990* Δ*Rmet_2614::*pLO*lacZ*	This work
DN 969	AE104 Δ*Rmet_1099* Δ*Rmet_3990::*pLO*lacZ*	This work
DN 970	AE104 Δ*Rmet_1099 (folE_1B2)* Δ*Rmet_3990 (folE_1A)*	This work
DN 971	AE104 Δ*Rmet_1099 (folE_1B2)* Δ*Rmet_2614 (folE_1B1)*	This work

Supplementary Table S14. DNA Primers used.

Name/orientation	5'→3' sequence	position
control primer		
pBBR fwd ←	CGCAGTCGG CCTATTGGTTA	sequencing primer for pBBR MCS3 vectors
pBBR rev →	CGGCTCGTATGTTGTGTGGAA	sequencing primer for pBBR MCS3 vectors
pASK down →	AAAAGTGAAATGAATAGTTCGACAAAATC	sequencing primer for pASK-IBA3/IBA7 vectors
pASK up ←	CGGTAAACGGCAGACAAAAAAAATG	sequencing primer for pASK-IBA3/IBA7 vectors
pask_fwd_XbaI →	GTGAAATGAATAGTTCGACAAAA	binds upstream of MCS in pASK-IBA3/IBA7 cloning vector
lascZ ralsti fusion test →	CACAGATGAAACGCCGAGTTAACG	test/sequencing primer for pECD794.1 vectors, binds 278 bp upstream of ATG_{lacZ}
pLO2-lacZ-5519 ←	GGCGGAAAATCGTGTTGAGGC	test/sequencing primer for pECD794.1 vectors, binds binds 438 bp downstream of ATG_{lacZ}
pGEM fwd →	GCGAAAGGGGGATGTGCTGC	sequencing primer for pGEM T-Easy vectors
pGEM rev →	ATATGGTCGACCTGCAGGCG	sequencing primer for pGEM T-Easy vectors
Rmet_1098 lacZ test	TGT TGC CAC GCT CGT CCC TTT G	test primer, binds 260 bp downstream of ATG_{Rmet_1098}
gene disruption		
Disrup_1099_SphI →	AAAGCATGCCTACGCCTGCGTTTTGATC	binds 345 bp downstream of ATG_{Rmet_1099}
Disrup_1099_XbaI ←	AAATCTAGACCCCAGGTTCGGCAAATC	binds 675 bp downstream of ATG_{Rmet_1099}
lacZ_Dis_2614_SphI →	AAAGCATGCACCGCGCGCCACTGGAC	binds 226 bp downstream of ATG_{Rmet_2614}
lacZ_dis_2614_XbaI ←	AAATCTAGAGCACCAGCGCCTCGACATC	binds 563 bp downstream of ATG_{Rmet_2614}
lacZDis_3990_SphI →	AAAGCATGCGTATTACGCACGCTGGTGATC	binds 186 bp downstream of ATG_{Rmet_3990}
lacZ-dis_3990_XbaI ←	AAATCTAGAGACCTGTGCCACGGCTTCTT	binds 531 bp downstream of ATG_{Rmet_3990}
lacZ-fusions		
lacZFus_3990_SalI →	AAAGTCGACCACCTGTGCCCGATCAT	binds 332 bp upstream of TGA_{Rmet_3990}
lacZFus_3990_XbaI ←	AAATCTAGATCAGCGGCGATTGAGATTCAG	binds directly upstream of TGA_{Rmet_3990}
lacZ_fus_1099_XbaI ←	AAATCTAGATCATGCGGCCACCTCCC	binds directly upstream of TGA_{Rmet_1099}
lacZ_fus_1099_PstI →	AAACTGCAGAGTGAGGCGTCGATCAC	binds 658 bp downstream of ATG_{Rmet_1099}

lacZ_fus_2614_XbaI ←	AAATCTAGATTACTGATGCCGCTTGTCGT	binds directly upstream of TAA_{Rmet_2614}
lacZ_fus_2614_PstI →	AAACTGCAGTGCCCGTGCTCGAAGAAG	binds 455 bp downstream of ATG_{Rmet_2614}
glyA_lacZ_fus_PstI →	AAACTGCAGGCCACGTGATGCTGGTGGAC	binds 940 bp downstream of ATG_{Rmet_2680}
glyA_lacZ_fus_XbaI ←	AAATCTAGATCAGCCGTAGACCGGGAAG	binds directly upstream of TGA_{Rmet_2680}
cre-lox		
CreLox_F1_1099_MunI →	AAACAATTGGGCGCCGTTGCCAGGCAC	binds 271 bp upstream of ATG_{Rmet_1099}
CreLox_F1_1099_NcoI ←	AAACCATGGCATGGGGAGCCCAAAGTGT	binds directly upstream of ATG_{Rmet_1099}
CreLox_F2_1099-ApaI →	AAAGGGCCCGCCGCATGACTTCCGCGC	binds directly downstream of TGA_{Rmet_1099}
CreLox_F2_1099-AgeI ←	AAAACCGGTACGCGCGCATGTCTTCGTGG	binds 293 bp downstream of TGA_{Rmet_1099}
CreLox_F1_2614_MunI →	AAACAATTGGGCGCCGTTGCCAGGCAC	binds 296 bp upstream of ATG_{Rmet_2614}
CreLox_F1_2614_NcoI ←	AAACCATGGCATGGGGAGCCCAAAGTGT	binds directly upstream of ATG_{Rmet_2614}
CreLox_F2_2614-ApaI →	AAAGGGCCCGCCGCATGACTTCCGCGC	binds directly downstream of TAA_{Rmet_2614}
CreLox_F2_2614-AgeI ←	AAAACCGGTACGCGCGCATGTCTTCGTGG	binds 291 bp downstream of TAA_{Rmet_2614}
CreLox_F1_3990_MunI →	AAACAATTGCGCCTCTGATACCGTCAC	binds 309 bp upstream of ATG_{Rmet_3990}
CreLox_F1_3990_NcoI ←	AAACCATGGGTGTGGCCGCAGGGAG	binds directly upstream of ATG_{Rmet_3990}
CreLox_F2_3990_ApaI →	AAAGGGCCCCTCGCTGCCCGGAGGGAACA	binds directly downstream of TGA_{Rmet_3990}
CreLox_F2_3990_AgeI ←	AAAACCGGT-CGTTGACGCGTCCCATCGAC	binds 312 bp downstream of TGA_{Rmet_3990}
Heterologous expression in *E.coli*		
pASK_Rmet_1099_SacI →	AAAGAGCTCCGCCCAGGACATTGGGGATC	binds directly downstream of ATG_{Rmet_1099}
pASK3_Rmet_1099_NcoI ←	AAACCATGGGCGGCCACCTCCCGTGAATG	binds directly upstream of TGA_{Rmet_1099}
pASK_2614_EcoRI →	AAAGAATTCAATGACATCAATCCCGCCTTC	binds directly downstream of ATG_{Rmet_2614}
pASK_2614_NcoI ←	AAACCATGGCA-CTGATGCCGCTTGTCGTG	binds directly upstream of TGA_{Rmet_2614}
pASK_3990_XhoI →	AAACTCGAGTCACACAAGGATCAGTCCGTC	binds directly downstream of ATG_{Rmet_3990}
pASK_3990_NcoI ←	AAACCATGGACGCGGCGATTGAGATTCAC	binds directly upstream of TGA_{Rmet_3990}

Supplementary Figure S1. Maps of genetic regions encoding *folE* genes. Maps are shown of determinants in the indicated regions with NPKM values (nucleotide activities per kilobase of exon model per million mapped reads) on one DNA strand (red) or the other direction of transcription (blue) in *C. metallidurans* CH34 cells cultivated without added metals or chelators (M1 medium). Above are the Rmet locus and gene names, the mean NPKM and response values (7). TSSs (transcriptional start points, flags) are indicated with the corresponding TSS score, white for scores < 50 with no promoter consensus motifs indicated, red shades for strong (>100, red), or weak (50-100, orange) RpoD promoters, blue shades (strong, medium, light) accordingly if not associated to the RpoD model. The TSS determination and association of the TSS to RpoD are published (5).

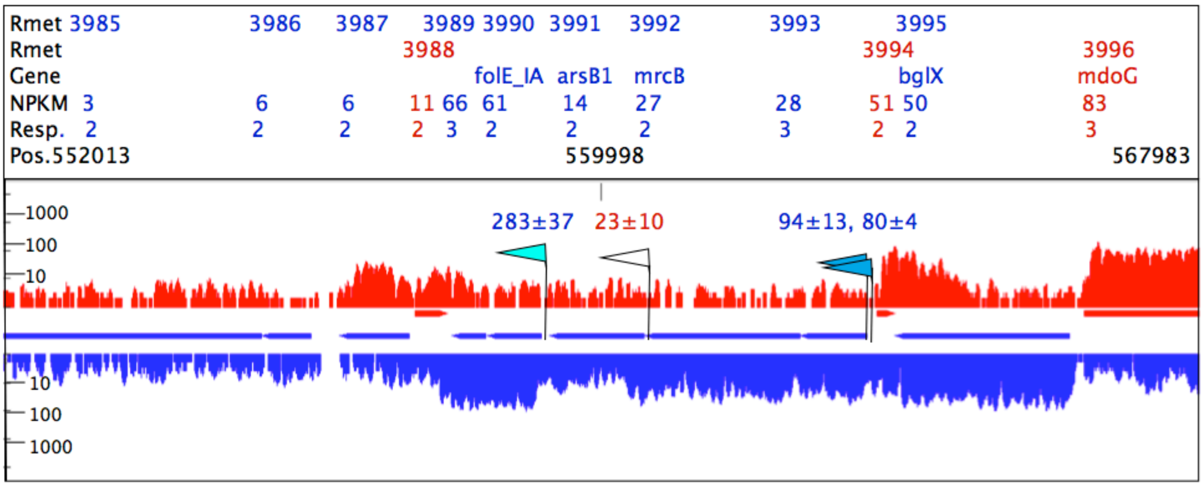

Panel A. The genes surrounding *folE_IA.*

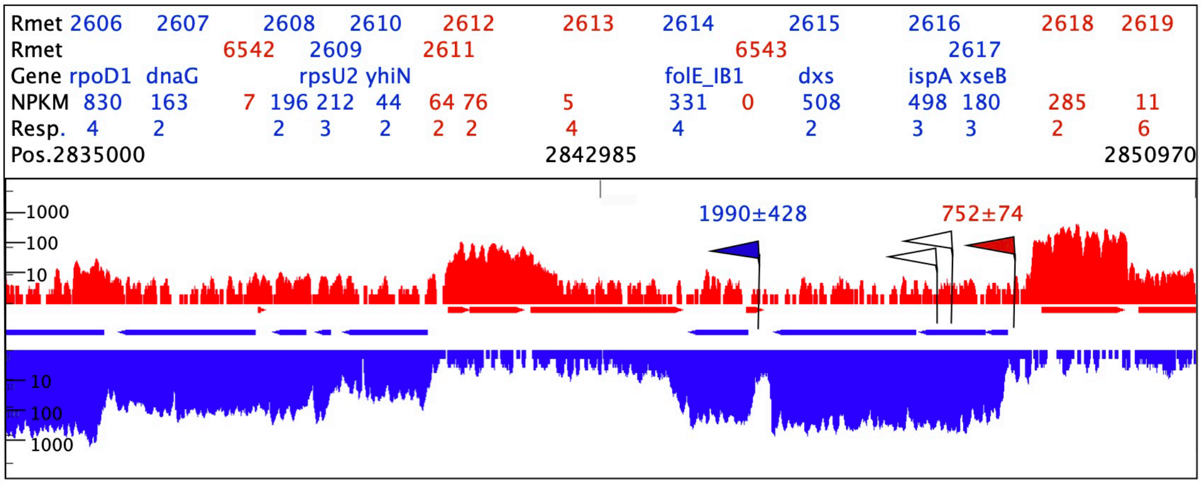

Panel B. The genes surrounding *folE_IB1.*

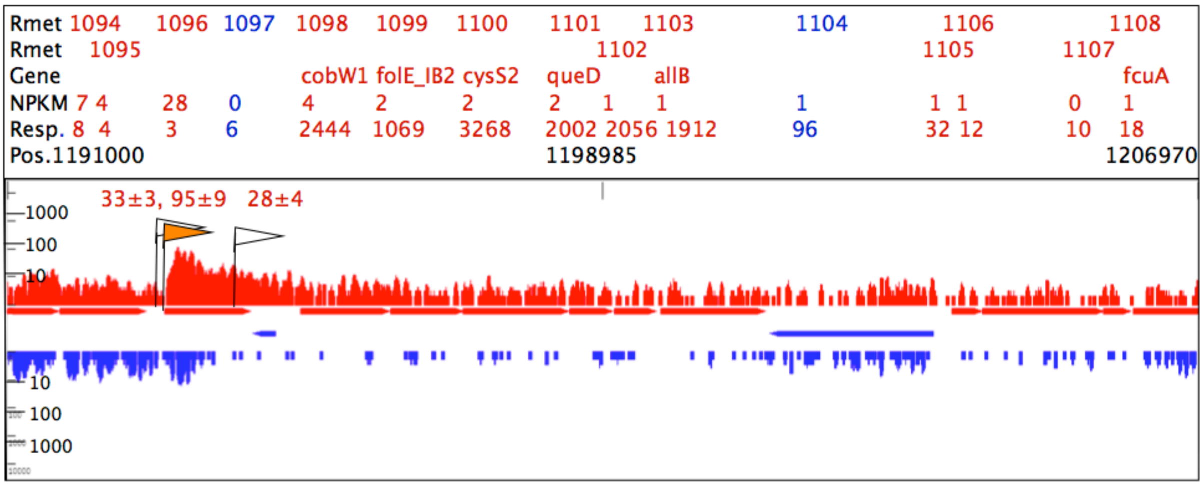

Panel C. The operon Op0317f_1 that contains *folE_IB2.*

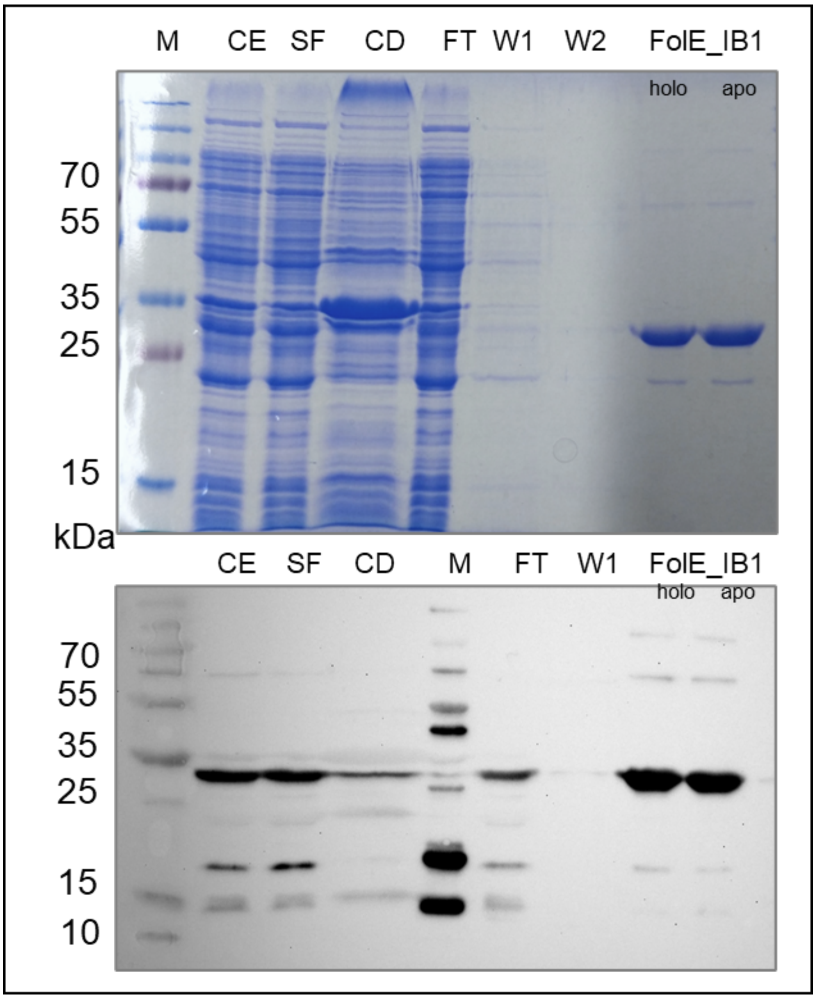

Supplementary Figure S2. Purification of FolE_IB1(Rmet_2614). The gene was cloned in plasmid pASK-IBA3+ and the *E. coli* strain Rosetta^{TM} (DE3) pLysS was transformed. Cells were cultivated in LB at 37 °C with shaking and the expression of *folE_IB1* was induced with 200 µg/L anhydrotetracycline. The soluble fraction (SF) was applied to a streptactin column after the separation from the crude extract (CE). The flow-through (FT) and two washing fractions (W1, W2) were collected. The protein was eluted with 25 mM biotin, collected and concentrated (FolE_IB1 holo). After the incubation with EDTA (100x excess), EDTA was removed by the desalting column and FolE_IB1 was concentrated (apo).

CE, SF, CD (cell debris), FT: 25 µg of the total protein loaded; W1 + W2: 25 µl; FolE_IB1 holo + apo: 5 µg

Top: 12.5% SDS PAGE, Coomassie Blue stain

Bottom: Strep-tag detection using Strep-Tactin HRP conjugate

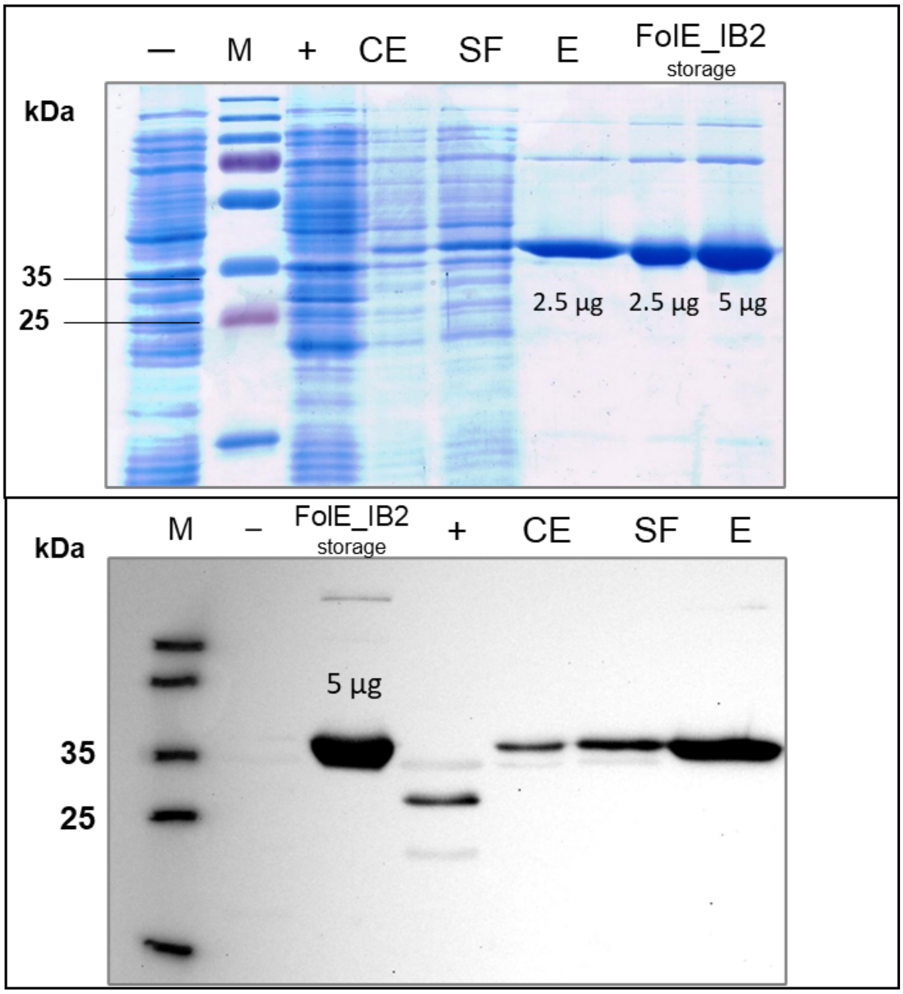

Supplementary Figure S3. Purification of FolE_IB2(Rmet_1099). The same protocol as for FolE_IB1 was used.

20 µg dry mass of the control (-) and induced (+) cells were applied on the gel. 25 µg (total protein) of crude extract (CE) and soluble fraction (SF) were applied. 2.5 µg or 5 µg of combined elution fractions (E) or purified FolE_IB2 (storage), respectively, after addition of glycerol for the storage were investigated.

Top: 12.5% SDS PAGE, Coomassie Blue stain

Bottom: Strep-tag detection using Strep-Tactin HRP conjugate

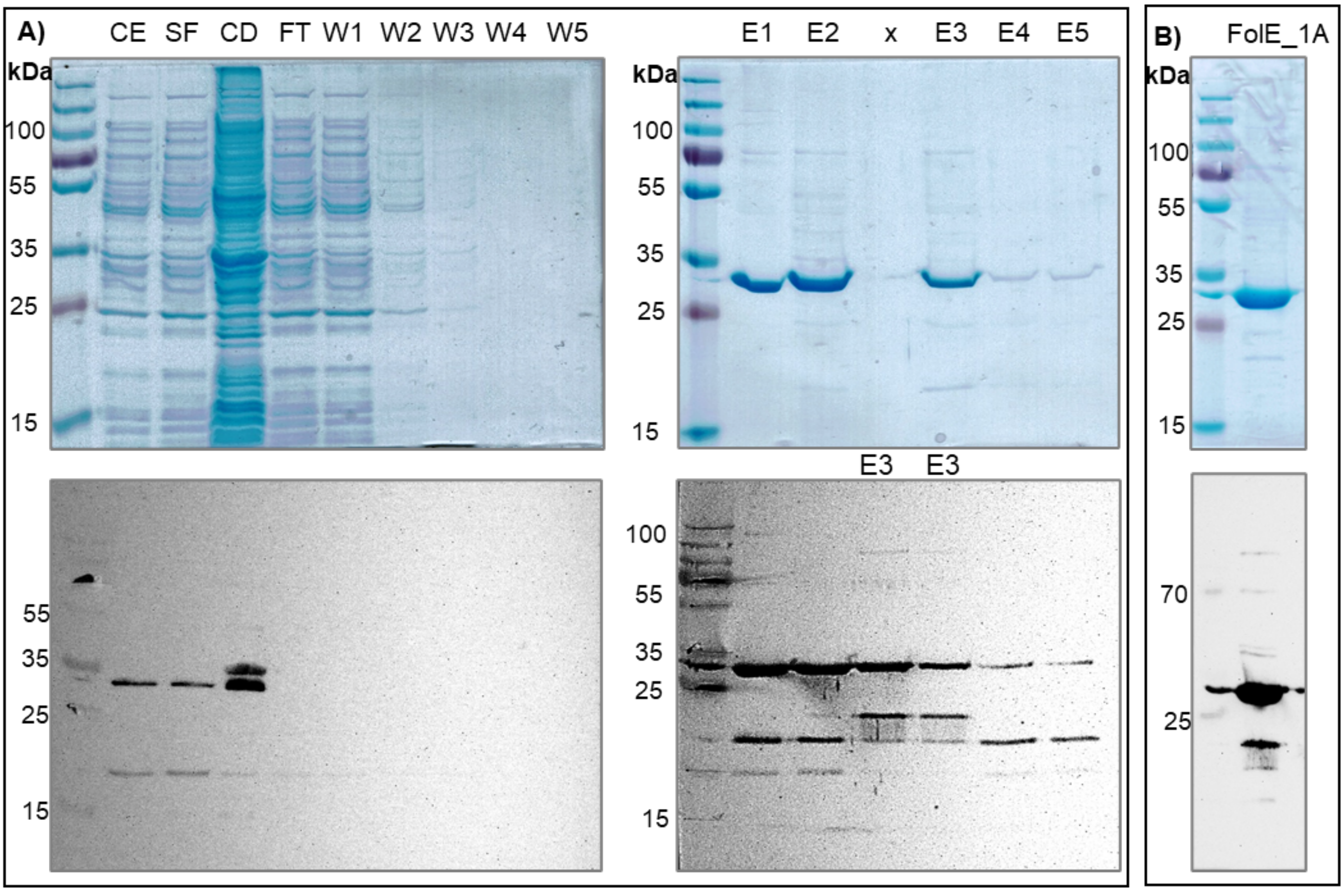

Supplementary Figure S4. Purification of FolE_IA(Rmet_3990). The same purification protocol as for FolE_IB1 was used.

A) 25 µg (total protein) of crude extract (CE), soluble fraction (SF), cell debris (CD) and the washing fraction W1 were applied. For the following steps (washing W2-W5 and elution E1-E5), 25 µl of the sample were put on the gel.

B) Combined elution fractions after concentration with Vivaspin, 8 µg of the purified FolE_IA were applied.

Top: 12.5% SDS PAGE, Coomassie Blue stain

Bottom: Strep-tag detection using Strep-Tactin HRP conjugate

```
2912761 CAGCAAGTTG CGAGCCAATC CTCGCCAGGC CTTGCCGGAT CAGGGCCGGG CGCACATCCG

2912821 GGCCGGCCAT CGTCTTGCAG CAAGCCGCTT GCTCGCGTAA ACTCTGCGGC AACCATTCCA
                        -35                               -10          TSS   pfl

2912881 CCTTGCGCGA CTGGCGAAGT CAGTGGGTAC TACCACGAGG AAGCGCAAGT TCATTGGGCG
          M  R  D   W  R  S   Q  W  V   L  P  R  G   S  A  S   S  L  G
          glyA

2912941 TGATATTCGA TGCGCCTGCC GCTCGCCTGG GCAGCGAATG GGAAGCAGGG TGCGCCCTGC
        V  I  F  D   A  P  A   A  R  L   G  S  E  W   E  A  G   C  A  L
```

Supplementary Figure S5. The 5' end of the *glyA* gene with the *pfl* riboswitch. The region of GenBank entry CP000352.1 (chromosome of *C. metallidurans*) is shown from base pair 2,912,761 to 2,913,000. The experimentally determined transcriptional start site TSS is shown with the corresponding -35 and -10 region of a RpoD-dependent promoter (5). The *pfl* sequence is in bold letters, the amino acid sequence of *glyA* indicated. This annotated beginning of *glyA* may not be correct since similarities with GlyA from *E. coli* start at amino acid position 97, so that the real translation initiation probably begins with an ATG and Met94.

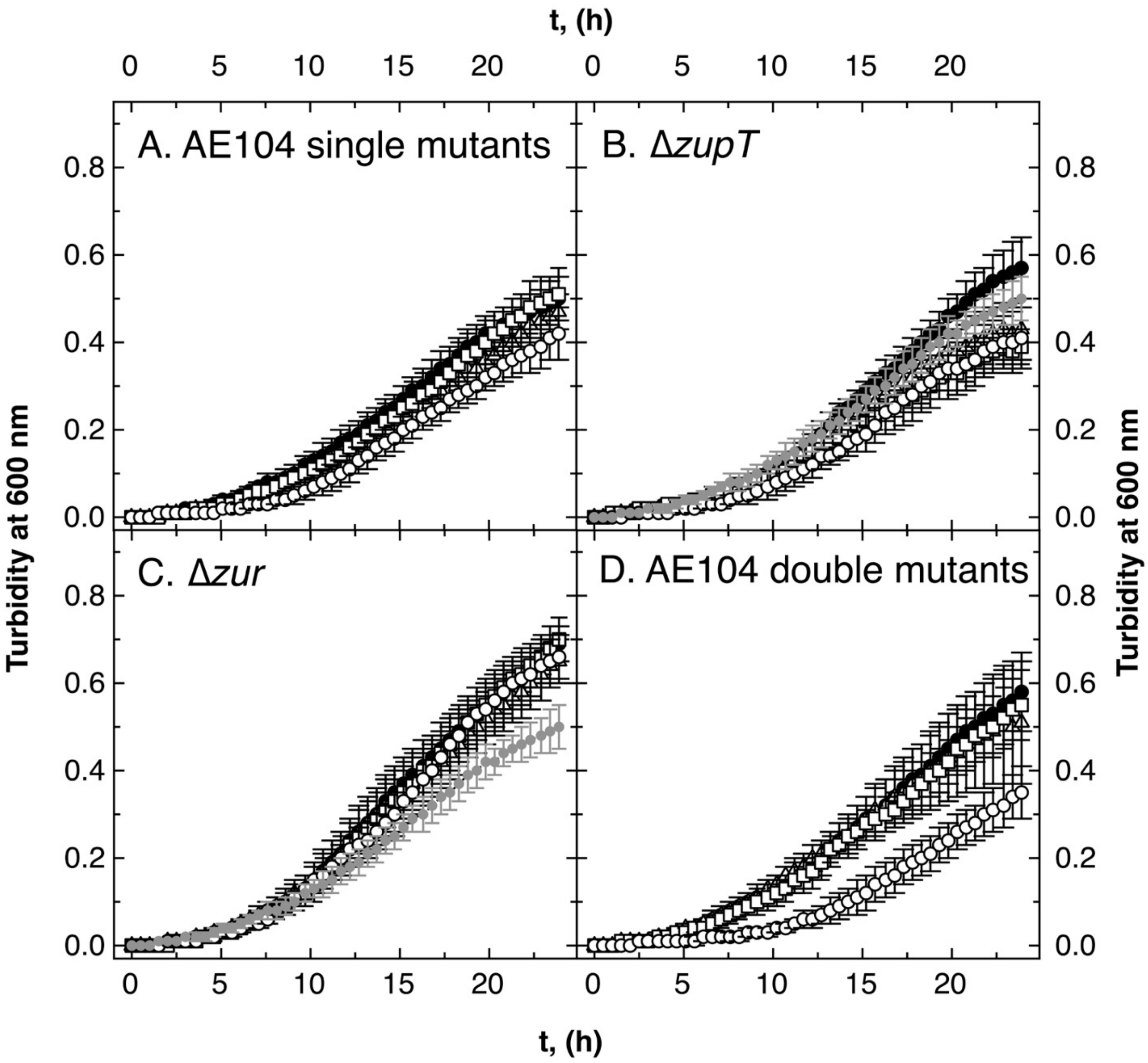

Supplementary Figure S6. Growth of ∆*folE* mutants with different backgrounds under iron starvation conditions. Growth of the *C. metallidurans* strains in low iron medium (M3, no SL6, no iron) in 96-well plates is shown. Panel A, parent background AE104; Panel B, ∆*zupT*; Panel C, ∆*zur*. Strain AE104 (closed black circles ● in Panel A, closed grey circles ● in Panels B and C), parent ∆*zupT* (closed black circles ● in Panel B), parent ∆*zur* (closed black circles ● in Panel C). Mutants in the respective background ∆*folE_IB2* (open triangles, ∆), ∆*folE_IB1* (open squares, ☐) and (open circles, ○). Panel D shows double mutants in the AE104 (closed black circles ●) background. Double mutants are ∆*folE_IB2* ∆*folE_IA* meaning B1 only (open triangles, ∆), ∆*folE_IB2* ∆*folE_IB1* or A only (open squares, ☐), ∆*folE_IA* ∆*folE_IB1::disrupted* or B2 only (open circles, ○), N > 3, deviations shown.

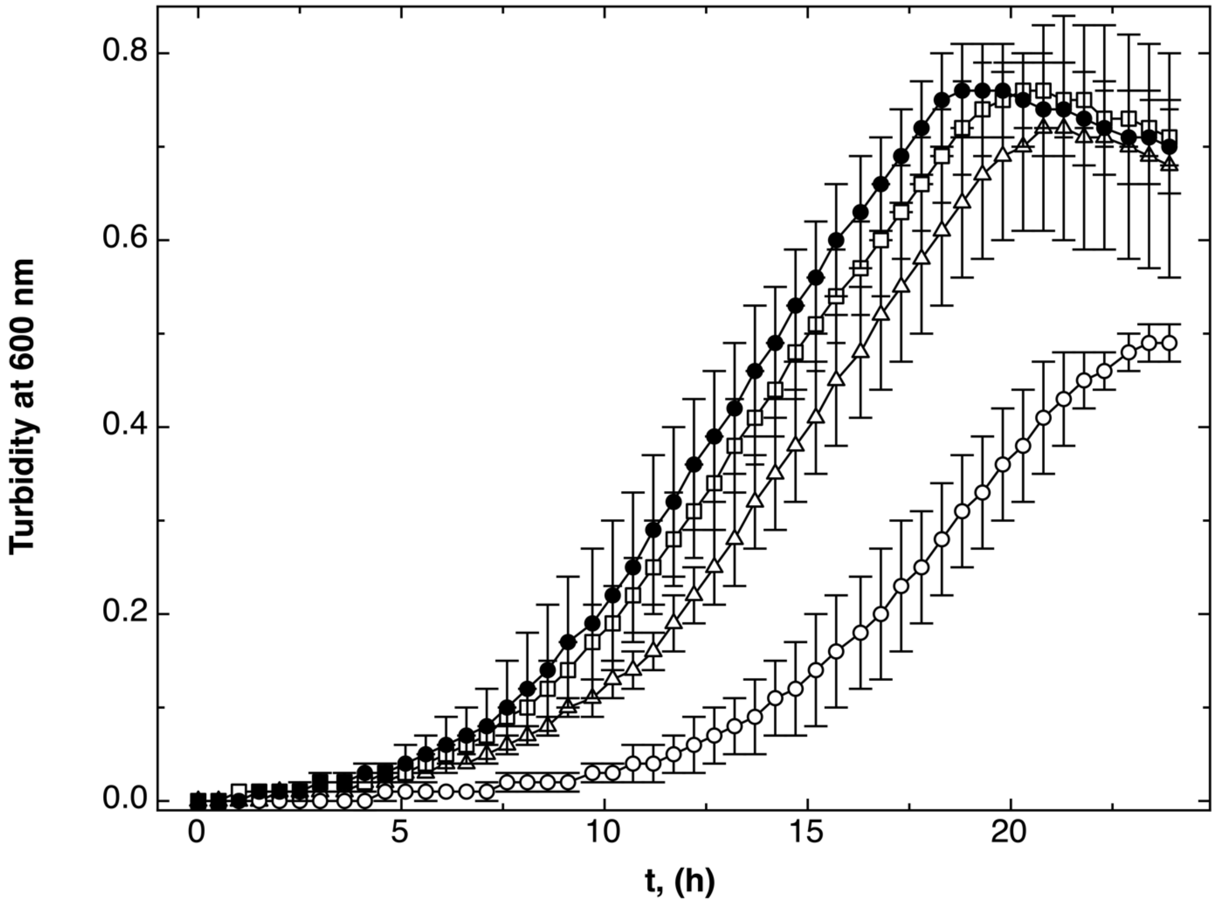

Supplementary Figure S7. Growth of the double Δ*folE* mutants with AE104 background in low iron zinc medium supplied with 0.5 µM iron. Growth of the *C. metallidurans* double mutant strains in low iron medium (M3, no SL6, no added iron) supplemented with 0.5 µM iron ammonium citrate in 96-well plates is shown**.** Strain AE104 (closed black circles, ●), its double mutants Δ*folE_IB2* Δ*folE_IA* meaning B1 only (open triangles, Δ), Δ*folE_IB2* Δ*folE_IB1* or A only (open squares, □), Δ*folE_IA* Δ*folE_IB::disrupted* or B2 only (open circles, ○), N > 3, deviations shown.

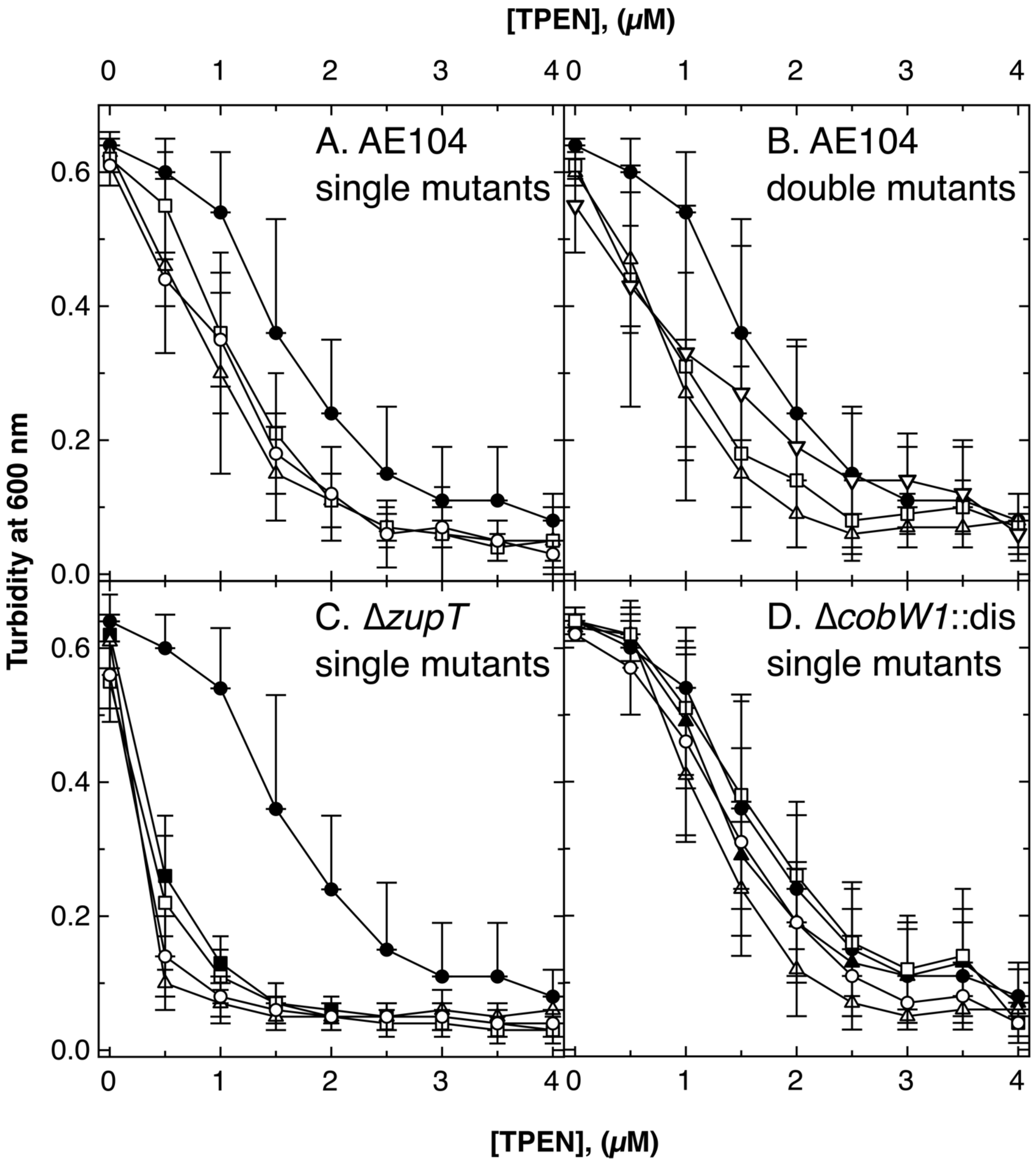

Supplementary Figure S8. Growth of Δ*folE* mutants in the presence of TPEN (*N*,*N*,*N'*,*N'*-tetrakis(2-pyridinylmethyl)-1,2-ethanediamine). Final turbidity of *C. metallidurans* strains in medium zinc TMM containing varying concentrations of TPEN is shown to derivatives of strain AE104 single (Panel A) and double (Panel B) mutants, Δ*zupT* single mutants (Panel C) and Δ*cobW1::disrupted* single mutants (Panel D). For the single mutants, strains are AE104 parent (closed circles, ●) shown in all panels, Δ*zupT* (closed squares, ■), Δ*cobW1::disrupted* (closed triangles, ▲) and their respective single mutants Δ*folE_IB2* (open triangles, Δ), Δ*folE_IB1* (open squares, ☐) and Δ*folE_IA* (open circles, ○). In panel B, the double mutants were Δ*folE_IA* Δ*folE_IB1::disrupted* (open inverted triangles, ▽), Δ*folE_IB2* Δ*folE_IA* (open triangles, Δ) and Δ*folE_IB2* Δ*folE_IB1* (open squares, ☐). N > 3, deviations shown.

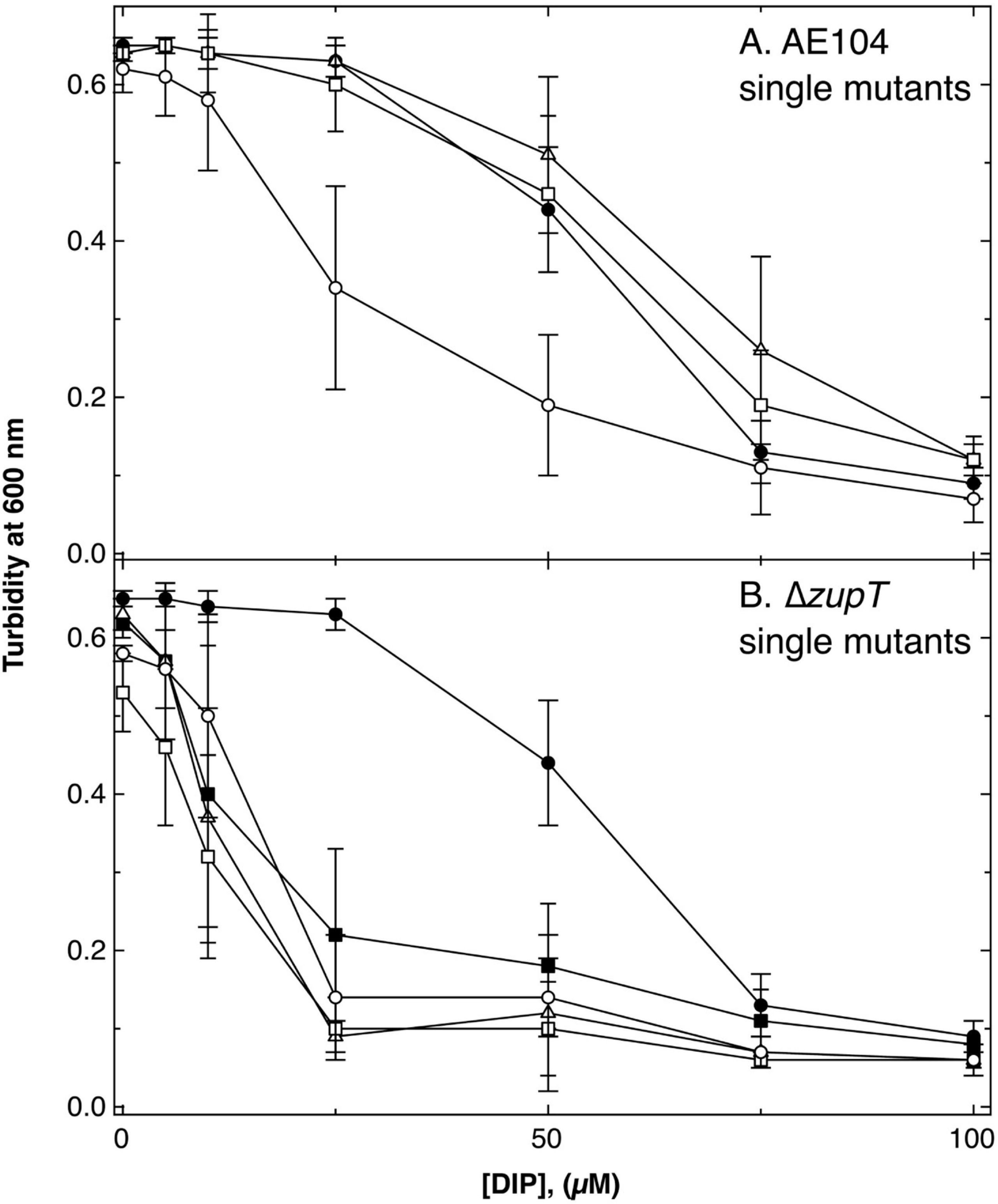

Supplementary Figure S9. Growth of Δ*folE* single mutants in the presence of DIP (2, 2' dipyridyl). Final turbidity of *C. metallidurans* strains in medium zinc TMM containing varying concentrations of DIP is shown to derivatives of strain AE104 (Panel A) and Δ*zupT* single mutants (Panel B). Strains are AE104 parent (closed circles, ●) shown in both panels, Δ*zupT* (closed squares, ■), and their respective single mutants Δ*folE_IB2* (open triangles, Δ), Δ*folE_IB1* (open squares, □) and Δ*folE_IA* (open circles, ○). N > 3, deviations shown.

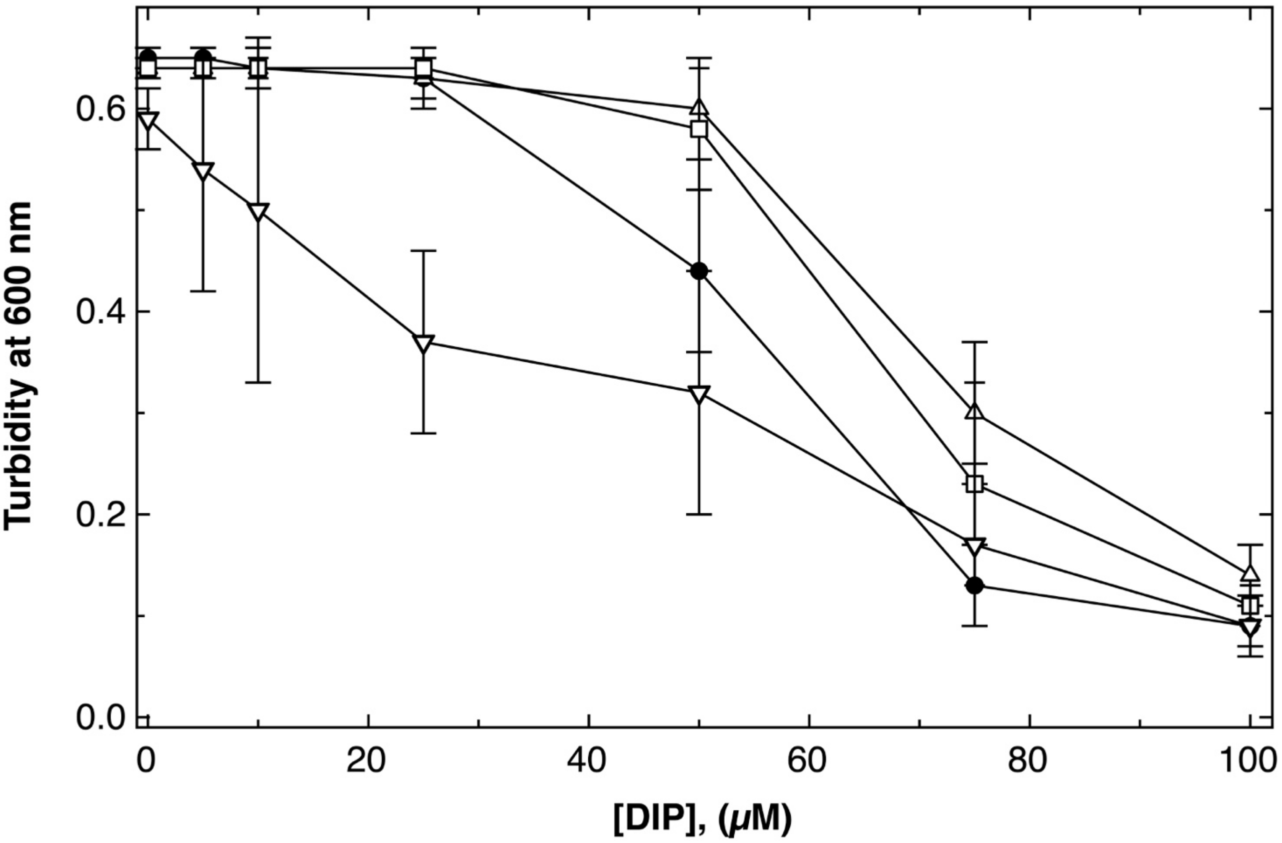

Supplementary Figure S10. Growth of ∆*folE* double mutants in the presence of DIP (2, 2' dipyridyl). Final turbidity of *C. metallidurans* strains in medium zinc TMM containing varying concentrations of DIP is shown. Strains are AE104 parent (closed circles, ●) and the double mutants were ∆*folE_IA* ∆*folE_IB1::disrupted* (open inverted triangles, ▽), ∆*folE_IB2* ∆*folE_IA* (open triangles, ∆), and ∆*folE_IB2* ∆*folE_IB1* (open squares, ☐). N > 3, deviations shown.

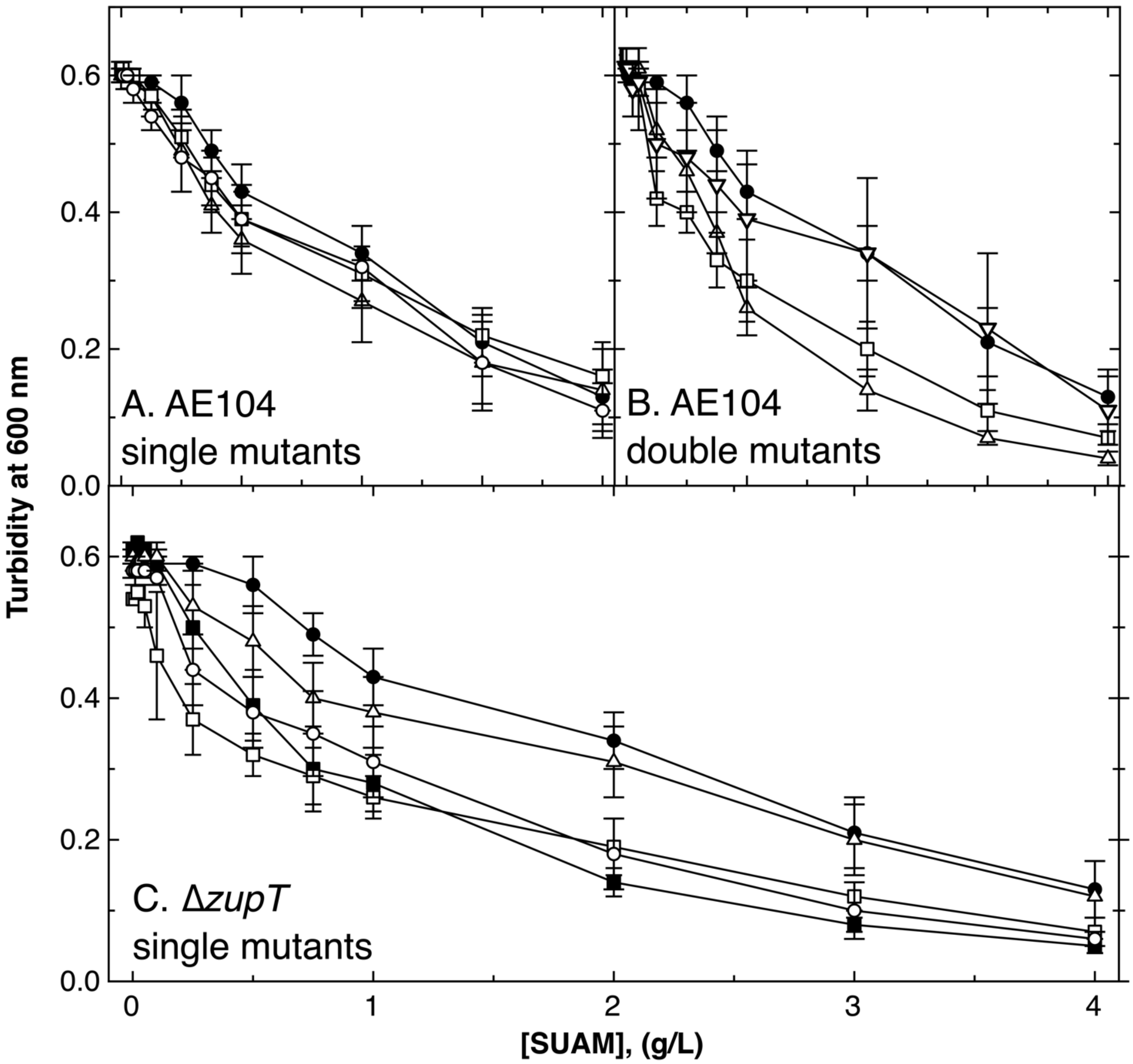

Supplementary Figure S11. Growth of Δ*folE* mutants in the presence of sulfonamide. Final turbidity of *C. metallidurans* strains in medium zinc TMM containing varying concentrations of sulfonamide (SUAM) is shown to derivatives of strain AE104 single (Panel A), double (Panel B), and Δ*zupT* single mutants (Panel C). For the single mutants, strains are AE104 parent (closed circles, ●) shown in all panels, Δ*zupT* (closed squares, ■), and their respective single mutants Δ*folE_IB2* (open triangles, Δ), Δ*folE_IB1* (open squares, □) and Δ*folE_IA* (open circles, ○). In panel B, the double mutants were Δ*folE_IA* Δ*folE_IB1::disrupted* (open inverted triangles, ▽), Δ*folE_IB2* Δ*folE_IA* (open triangles, Δ) and Δ*folE_IB2* Δ*folE_IB1* (open squares, □). N > 3, deviations shown.

Query 5K9G-pdb, protein from *Neisseria gonorrhoeae*
FolE_IB-Alignment

Query mit FolE_IB1: Binding Cys153, His165, Glu207

```
Query  4    IADVQSSRDLRNLPINQVGIKDLRFPITLKTAEGTQSTVARLTMTVYLPAEQKGTHMSRF  63
            + DVQSS D R + I +VG+K +R+P+TLKT  G   TV    M V LPA+QKGTHMSRF
Sbjct  10   MPDVQSSVDTRQIVIQRVGVKGVRYPLTLKTPAGAVGTVGTFNMDVRLPADQKGTHMSRF  69

Query  64   VALMEQHTEVLDFAQLHRLTAEMVALLDSRAGKISVSFPFFRKKTAPVSGIRSLLDYDVS  123
            VAL+E++   LD A    L  +M+  L++ AG+I VSFP+F  KTAPVSG++SLLDY+V+
Sbjct  70   VALLEENRAPLDLAAFRALVDDMLVRLEADAGRIEVSFPYFITKTAPVSGVQSLLDYEVT  129

Query  124  LTGEMKDGAYGHSMKVMIPVTSLCPXSKEISQYGAHNQRSHVTVSLTSDAEVGIEEVIDY  183
            L   E +DG     M  ++PVTSLCP SK+ISQYGAHNQRSH+T+ +    ++ +E ++
Sbjct  130  LVAEARDGQTRMFMTALVPVTSLCPCSKKISQYGAHNQRSHITMRVELAGDLDVEALVRM  189

Query  184  VETQASCQLYGLLKRPDEKYVTEKAYENPKFVEDMVRDVATSLIADKRIKSFVVESENFE  243
             E +ASC+L+GLLKRPDEK+VTE+AYENPKFVED+VRD+A  L AD RI ++ +E+ENFE
Sbjct  190  AEEEASCELWGLLKRPDEKFVTERAYENPKFVEDLVRDIAMRLNADDRIVAYTLEAENFE  249

Query  244  SIHNHSAYAYIAY  256
            SIHNHSAYA I +
Sbjct  250  SIHNHSAYAVIEH  262
```

Query mit FolE_IB2: Binding Cys156, His200, Glu245

```
Query  17   PINQVGIKDLRFPITLKTAEGTQSTVARLTMTVYLPAEQ-KGTHMSRFVALMEQHTEVLD  75
            P+  VG++ +  PI L      +   AR  + V LPA + KG HMSR  AL++   +
Sbjct  19   PLEWVGMQGIDLPIRLVEPGYRRQLHARADIHVDLPAPRVKGIHMSRLYALLDALDDGEG  78

Query  76   FAQLHRLTAEMVALLDSR------AGKISVSFPFFRKKTAPVS-GIRSLLDYDVSLTGEM  128
             + + R+   + A++DS       + ++ + F    ++ A V+ GI     Y V L   +
Sbjct  79   LSPI-RIRQLLEAMVDSHRDCETGSARLRLRFDLLVRRPALVTEGIAGWKSYPVRLDASL  137

Query  129  KDGAYGHSMKVMIPVTSLCPXSKEIS----------------------------QYGA--  158
                +  + ++    +S CP S  ++                            Q+G
Sbjct  138  VGNVFALTARITAGYSSTCPCSAALTRQLVEQGFAKAFAHERRVDVARVSAWLRQHGTLA  197

Query  159  --HNQRSH--VTVSLTSD-AEVGIEEVIDYVETQASCQLYGLLKRPDEKYVTEKAYENPK  213
              H+QRS   +TV +  D   +G+ +++D++E      +   +KR DE+       +N
Sbjct  198  TPHSQRSEASITVDIAVDLPNLGLLDLVDHIERALGTPVQTAVKRADEQAFAALNGQNLM  257

Query  214  FVEDMVRDVATSLIADKRIKSFVVESENFESIHNHSAYAYIA  255
            FVED  R +  +L  D R  +  V   + ES+H H A A+ A
Sbjct  258  FVEDAARRIQVAL--DGRFANPRVHVRHLESLHPHDAVAWAA  297
```

```
Query: FolE_IA from C. metallidurans and FolE from E. coli
Zinc-binding site conserved C126, H128, H129, C199

Query  34   ETRKSLIAGHMTEIMQLLNLDLADD-SLMETPHRIAKMYVDEIFSGLDYANFPKITLIEN  92
            E  ++ +   M ++++ L +D+  D +  ET  R+AKMY+ EIF+G  YA  P +T   N
Sbjct  50   EALQAEVEARMEDVLRTLVIDVDQDHNTRETARRVAKMYLKEIFAG-RYAKAPDVTEFPN  108

Query  93   KMKVDEMVTVRDITLTSTCEHHFVTIDGKATVAYIPK--DSVIGLSKINRIVQFFAQRPQ  150
              +++E++ V   I + S C HH    I G    V  +P     ++IGLSK   R+ ++     RPQ
Sbjct  109  VEQLNELMIVGPIRVRSACSHHLCPIIGSLWVGVMPNRHSNLIGLSKYARLAEWIMCRPQ  168

Query  151  VQERLTQQILIALQTLLGTNNVAVSIDAVHYCVKARGIRDATSATTTTSLGGLFKSSQNT  210
            +QE    Q+   LQ  +   + +A+ ++A H+C+   RG+RD   +   T + + G F
Sbjct  169  IQEEAVAQVADLLQEKMNPDGLAIVMEAEHFCMHWRGVRDTDAKMTNSVMRGSFLKDDKL  228

Query  211  RHEFL  215
            R EFL
Sbjct  229  RREFL  233
```

Supplementary Figure S12. Alignment of the amino acid sequence of the FolEs from *C. metallidurans* with that from other bacteria. Figures based on a BLAST alignment (10). Conserved regions in red letter, metal ligands underlined.

References

1. Grosse C, Herzberg M, Schüttau M, Nies DH. 2016. Characterization of the Δ7 mutant of *Cupriavidus metallidurans* with deletions of seven secondary metal uptake systems. mSystems 1:e00004-16.
2. Herzberg M, Dobritzsch D, Helm S, Baginski S, Nies DH. 2014. The zinc repository of *Cupriavidus metallidurans*. Metallomics 6:2157-2165
3. Große C, Poehlein A, Blank K, Schwarzenberger C, Schleuder G, Herzberg M, Nies DH. 2019. The third pillar of metal homeostasis in *Cupriavidus metallidurans* CH34: Preferences are controlled by extracytoplasmic functions sigma factors. Metallomics 11:291-316.
4. Karp P, Weaver D, Paley S, Fulcher C, Kubo A, Kothari A, Krummenacker M, Subhraveti P, Weerasinghe D, Gama-Castro S, Huerta A, Muñiz-Rascado L, Bonavides-Martinez C, Weiss V, Peralta-Gil M, Santos-Zavaleta A, Schröder I, Mackie A, Gunsalus R, Collado-Vides J, Keseler I, Paulsen I. 2014. The EcoCyc Database. EcoSal Plus doi:doi:10.1128/ecosalplus.ESP-0009-2013:doi:10.1128/ecosalplus.ESP-0009-2013.
5. Große C, Grau J, Große I, Nies DH. 2022. Importance of RpoD- and non-RpoD-dependent expression of horizontally acquired genes in *Cupriavidus metallidurans*. Microbiol Spectr 10: 10.1128/spectrum.00121-22.
6. Mergeay M, Nies D, Schlegel HG, Gerits J, Charles P, van Gijsegem F. 1985. *Alcaligenes eutrophus* CH34 is a facultative chemolithotroph with plasmid-bound resistance to heavy metals. J Bacteriol 162:328-334.
7. Große C, Kohl T, Herzberg M, Nies DH. 2022. Loss of mobile genomic islands in metal resistant, hydrogen-oxidizing *Cupriavidus metallidurans*. Appl Environ Microbiol 88:e02048-21.
8. Chandrangsu P, Huang X, Gaballa A, Helmann JD. 2019. *Bacillus subtilis* FolE is sustained by the ZagA zinc metallochaperone and the alarmone ZTP under conditions of zinc deficiency. Mol Microbiol 112:751-765.
9. Nies DH. 2019. The ancient alarmone ZTP and zinc homeostasis in *Bacillus subtilis*. Mol Microbiol 112:741-746.
10. Altschul SF, Madden TL, Schaffer AA, Zhang J, Zhang Z, Miller W, Lipman DJ. 1997. Gapped BLAST and PSI-BLAST: a new generation of protein database search programs. Nucl Acid Res 25:3389-3402.
